# Chronic reduction of synaptic proteins in the epileptogenic lesion of patients with hippocampal sclerosis

**DOI:** 10.3389/fnmol.2025.1635852

**Published:** 2025-07-23

**Authors:** Akiko Oota-Ishigaki, Nami Suzuki, Keiya Iijima, Yutaro Takayama, Yuiko Kimura, Kotaro Hattori, Masaki Iwasaki, Takashi Hayashi

**Affiliations:** ^1^Biomedical Research Institute, National Institute of Advanced Industrial Science and Technology (AIST), Tsukuba, Japan; ^2^Department of Neurosurgery, National Center Hospital, National Center of Neurology and Psychiatry (NCNP), Kodaira, Japan; ^3^Medical Genome Center, National Center of Neurology and Psychiatry (NCNP), Kodaira, Japan; ^4^Molecular Biosystems Research Institute, National Institute of Advanced Industrial Science and Technology (AIST), Tsukuba, Japan

**Keywords:** epilepsy, synaptic proteins, glutamate receptor, phosphorylation, epileptogenesis

## Abstract

Disturbance of synaptic proteins in the epileptogenic lesion is considered the basis for drug-resistant focal epilepsy. However, details of these molecular changes remain unknown because brain tissues are typically uncollectable from live patients. Using surgically excised brain tissues from epileptogenic lesions of patients with hippocampal sclerosis, we biochemically studied quantitative alterations in synaptic protein expression and their posttranslational phosphorylation of synaptic proteins, including glutamate receptors, which are the major excitatory neurotransmitter receptors. Compared to less epileptogenic control regions, most patients exhibited reduced synaptic protein expression in the lesion and decreased *α*-amino-3-hydroxy-5-methyl-4-isoxazolepropionate (AMPA) receptor phosphorylation in the epileptogenic lesion, indicating an overall weakening of synapses in the chronic condition. These molecular disturbances may explain the clinically observed basal hypoactivity and hypometabolism in epileptogenic lesions and may function as a fundamental mechanism of epileptogenesis. Furthermore, a history of febrile seizures is associated with increased AMPA receptor phosphorylation, which correlates with the enhancement of excitatory synaptic strength and reduced thresholds of hyperexcitation.

## Introduction

The disturbance of protein expression and their modifications in the brains of patients with neurological disorders remains unknown because brain tissues are typically uncollectable from living patients for experimental purposes. The neurosurgical removal of the cerebral cortices around epileptogenic lesions is a well-established and effective therapy for patients with drug-resistant focal epilepsy, in whom severe seizures cannot be controlled even by proper and adequate treatments with multiple anti-seizure medications (ASMs) (Wiebe et al., 2001; Kwan et al., 2010; Brodie et al., 2012; Chen et al., 2018). These surgically excised brain specimens from live humans exceptionally enable us to reveal epileptic seizure-associated molecular changes *in vivo*. Surgically excised brain tissues in the past decades have been registered in the National Center of Neurology and Psychiatry (NCNP) BioBank (Iwasaki et al., 2022), which is available for studying epilepsy-related synaptic disturbances at the molecular level.^1^ The hippocampal sclerosis is the most frequent neuropathological alteration in patients with intractable temporal lobe epilepsy. Surgical removal of the epileptogenic lesion can provide a significant reduction in seizure frequency in the majority of patients with hippocampal sclerosis (Bote et al., 2008).

Major excitatory synaptic transmission in the mammalian brain is mediated by glutamate and its receptors, glutamate receptors (GluRs). Excitatory synaptic strength is largely controlled by the synaptic surface expression of *α*-amino-3-hydroxy-5-methyl-4-isoxazolepropionate (AMPA)-type GluRs (AMPA receptors), the main subtype of ionotropic GluRs. Alteration of AMPA receptor expression on the postsynaptic membrane through their interaction with many types of GluR-binding proteins can bidirectionally lead synaptic strength to enhanced or reduced excitatory synaptic function (Collingridge et al., 2004; Kessels and Malinow, 2009; Huganir and Nicoll, 2013). Appropriate quantitative control of synaptic AMPA receptor numbers is critical for basal synaptic transmission, synaptic plasticity, and higher brain function. Among the four AMPA receptor subunits (GluA1, 2, 3, and 4; also known as GluR1–4, GluRA–D, or GluRα1–4), GluA1 plays a dominant role during activity-dependent AMPA receptor insertion into synapses. At the same time, GluA2 is the primary determinant during endocytosis of AMPA receptors from synapses (Anggono and Huganir, 2012). AMPA receptor trafficking to and from synapses is dynamically regulated by the posttranslational protein phosphorylation of AMPA receptors (Anggono and Huganir, 2012; Jiang et al., 2006; Lussier et al., 2015). In these processes, the reversible phosphorylation and dephosphorylation cycle modulates the trafficking of AMPA receptors to or from the postsynaptic membrane (Derkach et al., 2007; Diering and Huganir, 2018). The *N*-methyl-d-aspartate (NMDA)-type ionotropic GluRs (NMDA receptors) also play a crucial role in synaptic plasticity and synaptogenesis, serving as a basis of higher brain functions.

Based on previous findings in rodent models, we further analyzed quantitative alterations in the expression and modification of AMPA receptors and other synaptic proteins, using surgical specimens obtained from drug-resistant epileptic patients with hippocampal sclerosis. Compared with less epileptogenic control regions, a significant decrease of synaptic protein expression and phosphorylation was detected in the hippocampal epileptogenic lesions of most patients.

## Materials and methods

### Resective epilepsy surgery

This study included 12 patients with drug-resistant mesial temporal lobe epilepsy due to hippocampal sclerosis who were treated with anterior temporal lobectomy and amygdalohippocampectomy between November 2002 and June 2020 at the National Center Hospital, NCNP, Tokyo, Japan. A comprehensive presurgical evaluation, including high-field magnetic resonance imaging, long-term video-electroencephalography, fluorodeoxyglucose positron emission tomography (PET), and neuropsychological testing, was performed in all patients. The diagnosis of mesial temporal lobe epilepsy with hippocampal sclerosis was confirmed based on the International League Against Epilepsy classification and definition of epilepsy syndromes (Riney et al., 2022). The etiology of epilepsy was determined based on histopathological diagnosis, radiological findings, and clinical history. Clinical information, including age at surgery, sex, side of surgery, age at epilepsy onset, and medical history, was collected retrospectively from our database (Supplementary Table 3).

### Specimens in the NCNP BioBank

Surgically excised brain tissues from the hippocampal lesion and co-instantaneous tissues from the anterior temporal neocortex of the temporal lobe tip were obtained from 12 patients with hippocampal sclerosis. The patient cohort has an East Asian genetic origin. In mesial temporal lobe epilepsy with hippocampal sclerosis, the mesial temporal region, including the hippocampus, is the most epileptogenic, and the anterior temporal neocortex, which can be surgically excised to gain access to the hippocampus, is less epileptogenic (Berg, 2008; Josephson et al., 2013). Thus, the hippocampal lesion and anterior temporal neocortex were assumed to be epileptogenic and less epileptogenic control tissues, respectively, in this study. All specimens were registered in the NCNP BioBank, and their comprehensive utilization was permitted for medical research. Surgically excised brain tissues were serially sliced into 5 mm-thick sections, followed by fixation or rapid freezing, alternately. Pathological diagnosis was confirmed in fixed slices by immunohistochemical staining. The corresponding regions in adjacent frozen tissue blocks with or without pathologically diagnosed hippocampal sclerosis were used for biochemical analysis (see details in Supplementary Figure 5).

All experiments were performed in accordance with the regulations and institutional guidelines of the NCNP and approved by the ethics committee (authorization number: NCNPBB-0125). The technical protocols for the experiments in this study were approved by the Institutional Review Committees of the National Institute of Advanced Industrial Science and Technology (authorization number: Hi2021-352).

### Antibodies

Anti-GluA1 (ab31232, ab183797, and ab109450), anti-GluA3 (ab40845), anti-GluA4 (ab109431 and ab109431), anti-GluA1pS831 (ab109464), anti-GluA1pS845 (ab76321), and anti-GluA2pS880 (ab52180) antibodies were purchased from Abcam (Cambridge, UK). Anti-GluA2 (cst13607), anti-GluN1 (cst5704), anti-GluN2A (cst4205), anti-GluN2B (cst14544), anti-GluA2pY876 (cst4027), anti-GluN2BpY1472 (cst4208), PSD-95 (cst3450), SAP102 (A7R8L and cst47421s), PICK1 (cst85325), SynGAP (D78B11 and cst5540), SAHNK3 (D5K6R and cst64555), Bassoon (D63B6 and cst6897), Synaptotagmin-1 (D33B7 and cst14558), Synaptophysin (D8F6H and cst36406), Synapsin-1 (D12G5 and cst5297), and GAPDH (cst2118) antibodies were purchased from Cell Signaling Technology (Danvers, MA, USA), GRIP1 (22398-1-AP) antibodies were purchased from Proteintech (Chicago, IL, USA).

### Protein expression and phosphorylation analysis

Tissue blocks from dissected frozen brain slices (Supplementary Figure 5) were rapidly pulverized with Multi-beads Shocker MB3000 (Yasui Kikai Co., Osaka, Japan) at 2,500 rpm for 15 s, cooling with liquid nitrogen. Pulverized specimens (3 mg) were lysed in 500-μL radio-immunoprecipitation assay (RIPA) buffer 10-mM Tris (pH 7.5), 150-mM NaCl, 1-mM EDTA, 1-mM NaF, 1-mM Na_3_VO_4_, 1% Triton X-100, 0.1% sodium dodecyl sulfate (SDS), 0.1% sodium deoxycholate, protease inhibitor cocktail (PIC; Roche, Basel, Switzerland) at 4°C. The adjusted protein extract samples (20 μg per lane) were separated by sodium dodecyl sulfate polyacrylamide gel electrophoresis (SDS-PAGE), followed by Western blotting with specific antibodies. Reactive bands were visualized using ImmunoStar LD (FUJIFILM Wako Pure Chemical Co., Osaka, Japan) with or without an enhancer or ECL Prime (Cytiva, Tokyo, Japan). Details are presented in the Supplementary Source Data. Chemiluminescent images were acquired using a chemiluminescence imaging system, LuminoGraph I (ATTO Co., Tokyo, Japan). Band intensities as well as their linearities were confirmed by LuminoGraph I. Protein amounts valuated from band signal intensities were quantified for AMPA receptor subunits GluA1–4, NMDA receptor subunits GluN1, GluN2A, and GluN2B, postsynaptic proteins PSD-95, SAP102, GRIP1, PICK1, SynGAP, or SHANK3, presynaptic proteins Bassoon, Synaptotagmin-1, Synaptophysin, or Synapsin-1, and GAPDH. Protein phosphorylations, valuated from band signal intensities, were quantified for GluA1pS831, GluA1pS845, GluA2pS880, GluA2pY876, and GluN2BpY1472. Changes in extremely faint bands on immunoblots were visually judged in case of difficulty in automatic detection by LuminoGraph I (Table 1B, Bassoon and Table 1C, SHANK3).

**Table 1:** Epilepsy-induced molecular changes in the living brain.

A	GluRs						
	AMPAR				NMDAR		
	GluA1	GluA2	GluA3	GluA4	GluN1	GluN2A	GluN2B
Up	33.3 (4/12)	33.3 (4/12)	33.3 (4/12)	16.7 (2/12)	0 (0/12)	16.7 (2/12)	0 (0/12)
Down	58.3 (7/12)	66.7 (8/12)	66.7 (8/12)	33.3 (4/12)	50 (6/12)	41.7 (5/12)	83.3 (10/12)
No change	8.3 (1/12)	0 (0/12)	0 (0/12)	0 (0/12)	0 (0/12)	0 (0/12)	0 (0/12)
Undetected	0 (0/12)	0 (0/12)	0 (0/12)	50 (6/12)	50 (6/12)	41.7 (5/12)	16.7 (2/12)

B	Presynaptic proteins			
	Bassoon	Synaptotagmin-1	Synaptophysin	Synapsin-1
Up	0 (0/12)	33.3 (4/12)	16.7 (2/12)	33.3 (4/12)
Down	50 (6/12)	50 (6/12)	75 (9/12)	50 (6/12)
No change	0 (0/12)	0 (0/12)	8.3 (1/12)	16.7 (2/12)
Undetected	50 (6/12)	16.7 (2/12)	0 (0/12)	0 (0/12)

C	Postsynaptic proteins					
	PSD95	SAP102	GRIP1	PICK1	SynGAP	SHANK3
Up	25 (3/12)	25 (3/12)	50 (6/12)	25 (3/12)	16.7 (2/12)	0 (0/12)
Down	66.7 (8/12)	58.3 (7/12)	50 (6/12)	75 (9/12)	58.3 (7/12)	50 (6/12)
No change	8.3 (1/12)	0 (0/12)	0 (0/12)	0 (0/12)	0 (0/12)	0 (0/12)
Undetected	0 (0/12)	16.7 (2/12)	0 (0/12)	0 (0/12)	25 (3/12)	50 (6/12)

D	GAPDH
Up	41.7 (5/12)
Down	33.3 (4/12)
No change	25 (3/12)
Undetected	0 (0/12)

E	GluR phosphorylation				
	AMPAR				NMDAR
	GluA1pS831	GluA1pS845	GluA2pS880	GluA2pY876	GluN2BpY1472
Up	25 (3/12)	8.3 (1/12)	0 (0/12)	0 (0/12)	8.3 (1/12)
Down	66.7 (8/12)	50 (6/12)	41.7 (5/12)	41.7 (5/12)	25 (3/12)
No change	8.3 (1/12)	8.3 (1/12)	0 (0/12)	0 (0/12)	0 (0/12)
Undetected	0 (0/12)	33.3 (4/12)	58.3 (7/12)	58.3 (7/12)	66.7 (8/12)

Percentage of each group in total patients (*n* = 12), classified by “up”- or “down”-regulated protein amounts or phosphorylation in the epileptogenic lesion. Alterations in the epileptogenic lesion for each patient were judged to go up or down by comparing with the control. Patients showing subtle differences (100 ± 5%) in each protein amounts or phosphorylation were classified into the “no change” group. The “undetected” group indicates patients who had unevaluable proteins because signals were undetected in the control tissue. The specific numbers of classified patients among the total patients are shown in parentheses as (*n*/12). **(A)** GluR protein expression: AMPA receptor subunits, GluA1, GluA2, GluA3, and GluA4; NMDA receptor subunits, GluN1, GluN2A, and GluN2B. **(B)** Presynaptic protein expression: Bassoon, Synaptotagmin-1, Synaptophysin, and Synapsin-1. **(C)** Postsynaptic protein expression: PSD-95, SAP102, GRIP1, PICK1, SynGAP, and SHANK3. **(D)** Ubiquitously expressed protein: GAPDH. **(E)** GluRs phosphorylation: GluA1pS831, GluA1pS845, GluA2pS880, GluA2pY876, and GluN2BpY1472.

### Statistical analysis

Statistical analyses were performed using Prism 10 (GraphPad Software, La Jolla, CA, USA) and Microsoft Excel. Bars in the figures are expressed as the mean ± standard error of the mean (SEM) unless indicated otherwise. Statistical tests conducted on each experiment are described in the figure legends.

## Results

### Epilepsy-induced reduction of synaptic protein expression

First, we performed a series of immunoblots with specific antibodies to test the alterations in presynaptic and postsynaptic protein amounts in the brains of patients with hippocampal sclerosis. Protein expression levels were examined in the brain tissue lysates from the hippocampal epileptogenic lesion and compared with those in the anterior temporal neocortex, which was prepared from the same living patient as the less epileptogenic control (Josephson et al., 2013). Sclerosis in the hippocampal epileptogenic lesion and no abnormality in the control region were pathologically confirmed. Judged only by qualitative comparison of protein expression levels between control and the epileptogenic lesions in each patient and classified by the number of patients whether they went up, down, or remained unchanged, GluRs (Table 1A and Supplementary Figures 1A,B), presynaptic proteins (Table 1B and Supplementary Figure 1C), and postsynaptic proteins (Table 1C and Supplementary Figure 1D) were downregulated in the majority of the patients. Percentage and classified patient numbers in total patients are shown in Table 1. The ubiquitously expressed protein GAPDH did not show a clear trend in expression (Table 1D).

**Figure 1:**
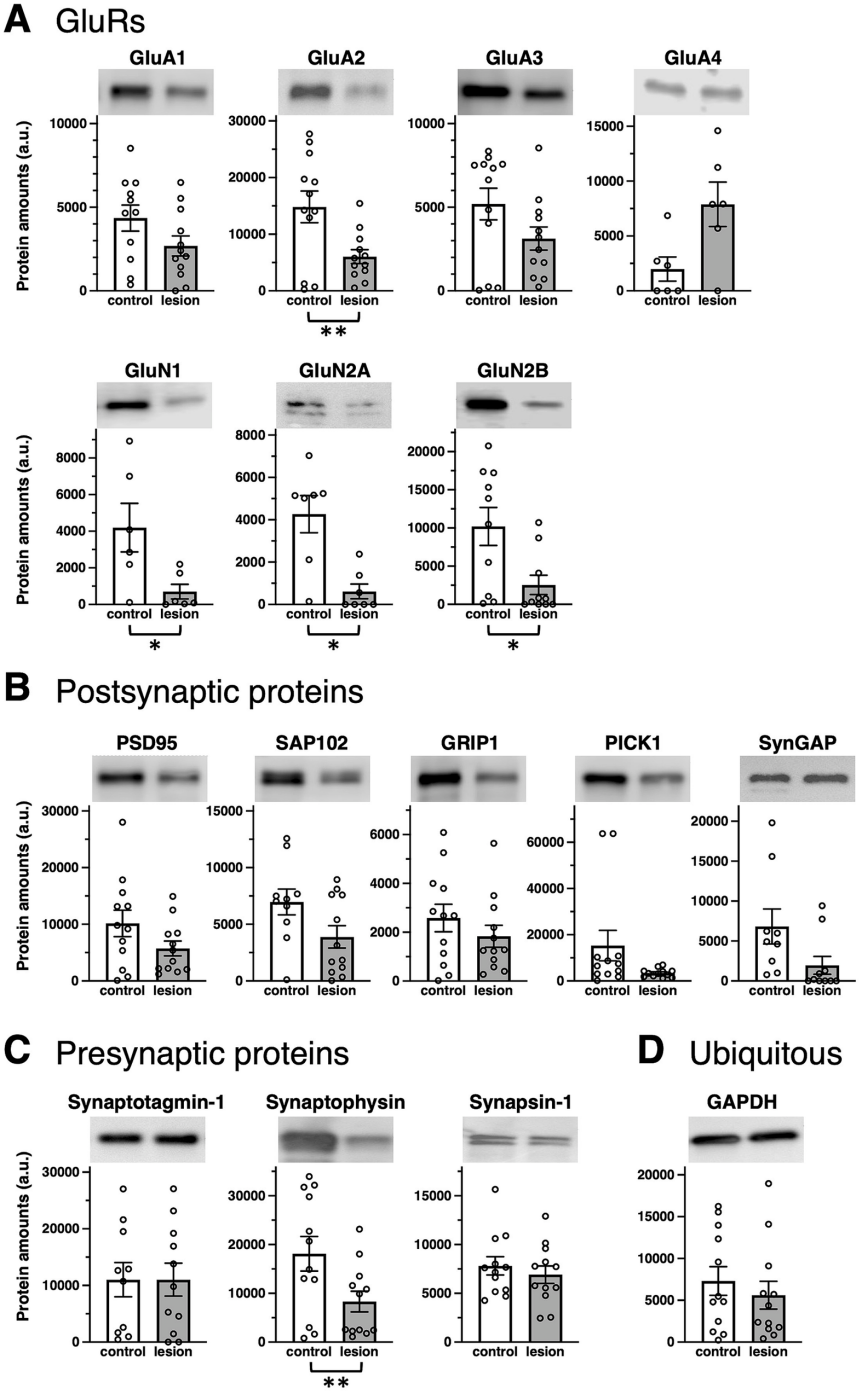
Epilepsy-induced changes of synaptic protein expression. Comparison of protein amounts between control (white bars) and epileptogenic lesion (gray bars) lysates. Protein amounts valuated from band signal intensities are shown in arbitrary units (a.u.) for each protein. Typical blots are shown as representative samples (top). **(A)** GluR protein expression: AMPA receptor subunits, GluA1 (*n* = 11, 12, respectively; *p* = 0.2172), GluA2 (*n* = 12, respectively; *p* = 0.0081), GluA3 (*n* = 12, respectively; *p* = 0.0661), and GluA4 (*n* = 6, respectively; *p* = 0.0656); NMDA receptor subunits, GluN1 (*n* = 6, respectively; *p* = 0.0395), GluN2A (*n* = 7, respectively; *p* = 0.0157), and GluN2B (*n* = 10, respectively; *p* = 0.0129). **(B)** Postsynaptic proteins: PSD-95 (*n* = 12, respectively; *p* = 0.1212), SAP102 (*n* = 10, 12, respectively; *p* = 0.2173), GRIP1 (*n* = 12, respectively; *p* = 0.2731), PICK1 (*n* = 12, respectively; *p* = 0.0947), and SynGAP (*n* = 9, 10, respectively; *p* = 0.1070). **(C)** Presynaptic proteins: Synaptotagmin-1 (*n* = 10, 11, respectively; *p* = 0.8574), Synaptophysin (*n* = 12, respectively; *p* = 0.0016), and Synapsin-1 (*n* = 12, respectively; *p* = 0.4740). **(D)** Ubiquitously expressed protein, GAPDH (*n* = 12, respectively; *p* = 0.3114). *p < 0.05, **p < 0.01, *t*-test.

Some significant differences were observed for synaptic proteins in average rate of changes of all four AMPA receptor subunits GluA1–4 (Figure 1A and Supplementary Figure 2A, top), NMDA receptor subunits GluN1, GluN2A, and GluN2B (Figure 1A and Supplementary Figure 2A, bottom), postsynaptic proteins such as GluRs-interacting proteins, postsynaptic density-95 (PSD-95), synapse-associated protein 102 (SAP102), glutamate receptor interacting protein 1 (GRIP1), protein interacting with C-kinase (PICK1), or synaptic Ras GTPase activating protein (SynGAP) (Figure 1B and Supplementary Figure 2B), and presynaptic proteins such as Synaptotagmin-1, Synaptophysin, and Synapsin-1 (Figure 1C and Supplementary Figure 2C). Among these synaptic proteins, GluA2, GluN1, GluN2A, GluN2B, and Synaptophysin showed significantly decreased protein amounts in epileptogenic lesions. The average protein amounts of GluA1, GluA3, PSD-95, SAP102, GRIP1, PICK1, and SynGAP in epileptogenic lesions also decreased to less than two-thirds of the control levels, but this difference was not statistically significant. The average protein amounts of GluA4 seemed to be increased in the epileptic lesion, but the difference was not statistically significant. Synaptotagmin-1 and Synapsin-1 showed no changes in expression. It is unclear whether the cause of some extreme outliers was a problem that occurred during surgery, sample storage, or due to each patient’s characteristics (Supplementary Figure 2). GAPDH exhibited little difference between the control and epileptogenic lesions (Figure 1D and Supplementary Figures 2D,E).

In individual patients, there was no apparent pattern for the expression of presynaptic proteins (Supplementary Figure 1C) and postsynaptic proteins (Supplementary Figure 1D). Some values increased or decreased, and vice versa.

### Epilepsy-induced changes in GluRs phosphorylation

**Figure 2:**
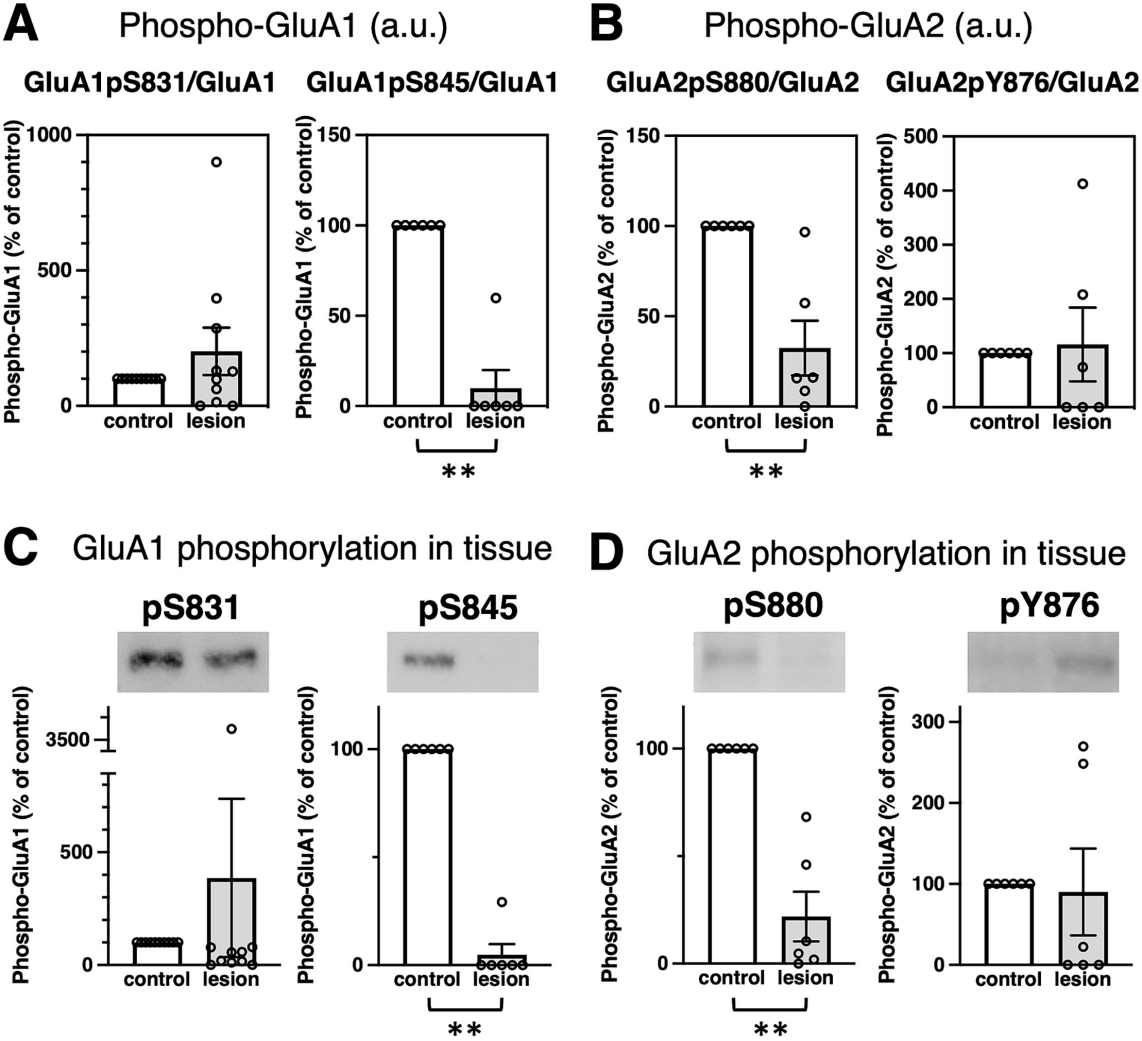
Epilepsy-induced changes of GluR protein phosphorylation. **(A,B)** Comparison of GluR phosphorylation between control (white bar, defined as 100%) and the epileptogenic lesions (gray bar) at each phosphorylation site. **(A)** GluA1 phosphorylation at Ser831 (GluA1pS831) was normalized to the GluA1 protein amounts (left, GluA1pS831/GluA1, 201.3 ± 87.66%, compared to control, *n* = 10, respectively; *p* > 0.9999). GluA1 phosphorylation at Ser845 (GluA1pS845) was normalized to GluA1 protein amounts (right, GluA1pS845/GluA1, 9.97 ± 9.97%, compared to control, *n* = 6, respectively; *p* = 0.0022). **(B)** GluA2 phosphorylation at Ser880 (GluA2pS880) was normalized to GluA2 protein amounts (left, GluA2pS880/GluA2, 32.42 ± 15.18%, compared to control, *n* = 6, respectively; *p* = 0.0022). GluA2 phosphorylation at Tyr876 (GluA2pY876) was normalized to GluA2 protein levels (right, GluA2pS876/GluA2, 115.84 ± 68.02%, compared to control, *n* = 6, respectively; *p* = 0.2987). **(C,D)** Comparison of protein phosphorylation amounts in total lysates between control (white bar, adjusted to 100%) and the epileptogenic lesions (gray bar). Typical blots are shown as representative samples (top). **(C)** GluA1 phosphorylation in whole tissue: GluA1pS831 (left, 386.6 ± 351.5%, compared to control, *n* = 10, respectively; *p* = 0.4359) and GluA1pS845 (right, 4.88 ± 4.88%, compared to control, *n* = 6, respectively; *p* = 0.0022). **(D)** GluA2 phosphorylation in whole tissue: GluA2pS880 (left, 21.90 ± 11.60%, compared to control, *n* = 6, respectively; *p* = 0.0022) and GluA2pY876 (right, 90.02 ± 53.61%, compared to control, *n* = 6, respectively; *p* = 0.2987). **p < 0.01, Mann–Whitney U test.

Next, we compared GluR phosphorylation between control and the epileptogenic lesions. Classified by the number of patients, the majority showed downregulation of GluR phosphorylation in the percentage of total patients (Table 1E). The major phosphorylation sites on the GluA1 subunit are Ser831 (GluA1pS831) and Ser845 (GluA1pS845). Based on rodent data, the phosphorylation of these sites was anticipated to increase in upregulated glutamatergic excitatory synapses (Anggono and Huganir, 2012; Diering and Huganir, 2018), whereas there was no significant difference in GluA1pS831 in the patient brain (Figures 2A,C, left and Supplementary Figure 3A). GluA1pS845 was significantly decreased in epileptogenic lesions (Figures 2A,C, right and Supplementary Figure 3B). Regarding GluA2 phosphorylation, our results showed reduced phosphorylation of GluA2 Ser880 (GluA2pS880) in the epileptogenic lesion (Figures 2B,D left and Supplementary Figure 3C). In contrast, phosphorylation of GluA2 at Tyr876 (GluA2pY876) in the epileptogenic lesion was similar to that in the control (Figures 2B,D, right and Supplementary Figure 3D). Both the AMPA receptor phosphorylation ratio, which was normalized to GluA protein expression levels (Figures 2A,B), and total phosphorylated GluA proteins at each site in specimens, which were adjusted by wet weight of whole tissue (Figures 2C,D), showed similar results. The alteration pattern of these phosphorylation sites varied in each patient (Supplementary Figures 3A–E).

### Characteristics and clinicopathological features of patients

Finally, we statistically examined the relationship between the aforementioned molecular changes and patient characteristics (Supplementary Table 3). The patients in this study consisted of five men and seven women. The mean age at surgery was 33.75 years (range: 11–56 years). The age range of seizure onset varies from 4 to 31 years. The full-scale intelligence quotient (FSIQ) was evaluated in all patients before surgery. Almost all protein expression (Supplementary Table 1A–E) and phosphorylation (Supplementary Table 2) demonstrated little correlation with the age at surgery and onset, disease duration (from onset to surgery), storage periods of the specimen, or preoperative FSIQ. Exceptionally, protein amounts of GluA3 (*r* = −0.77, Supplementary Table 1A) and Synaptotagmin-1 (*r* = −0.80, Supplementary Table 1D) in the control anterior temporal neocortex and Synaptophysin in the hippocampal epileptogenic lesion (*r* = −0.72, Supplementary Table 1D) had high negative correlation coefficients with storage periods of the specimen. The ratio of phosphorylation of GluA1 Ser845 to GluA1 protein expression (GluA1pS845/GluA1) in the epileptogenic lesion, which was normalized to the control, had a high correlation coefficient with the age of onset (*r* = 0.77, Supplementary Table 2). The sampled hemisphere was not related to the molecular changes. No obvious psychiatric complications were observed in any of the patients.

**Figure 3:**
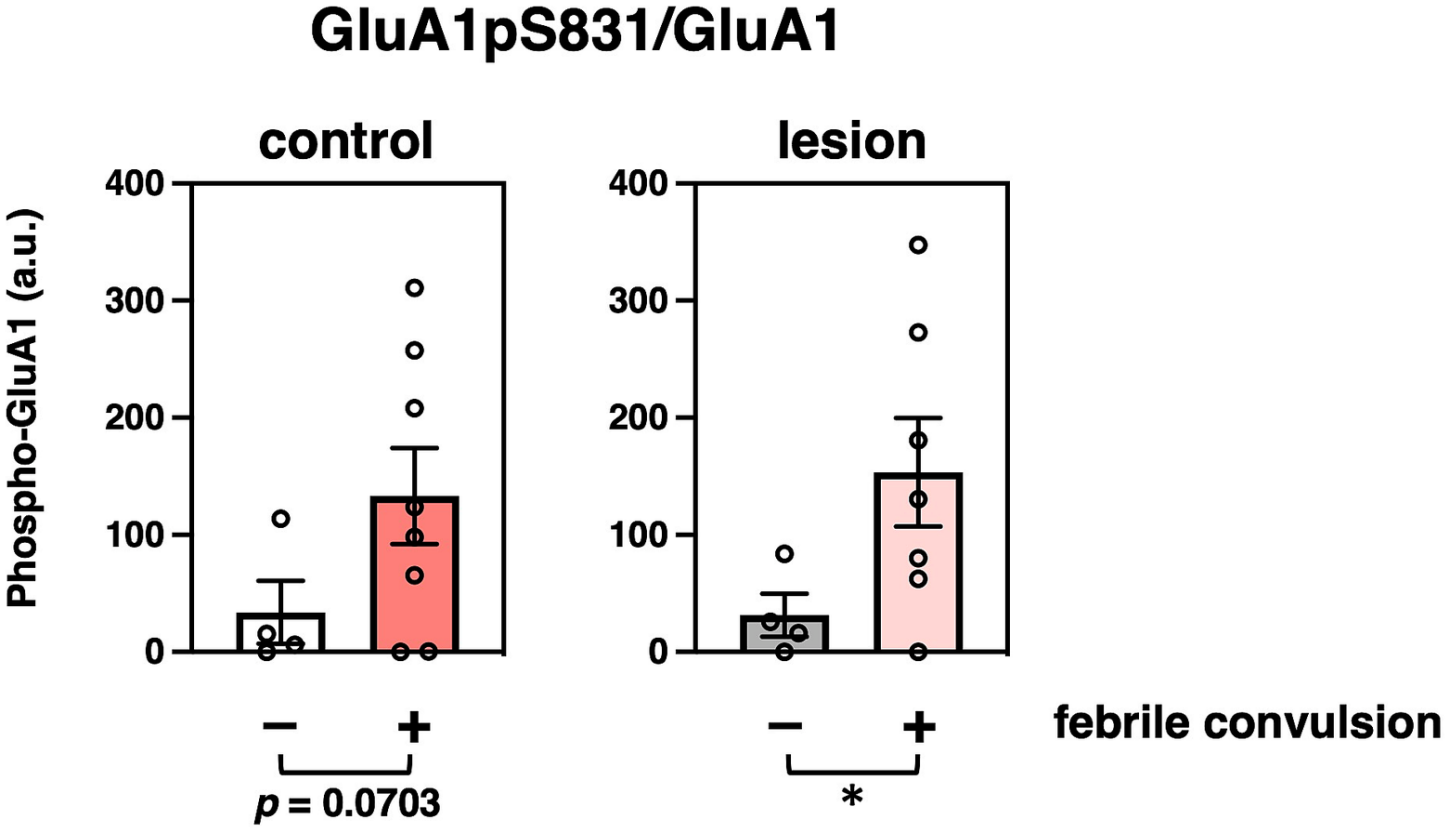
Effects of characteristics and clinicopathological features of patients on protein modifications. Comparison of GluA1 phosphorylation at Ser831 that were normalized to GluA1 protein amounts (GluA1pS831/GluA1) with (colored bar, *n* = 8) or without (white or gray bar, *n* = 4) previous febrile convulsion experiences in the control (left, compared to non-history, *n* = 4, 8, respectively, *p* = 0.0703) or epileptogenic lesion (right, compared to non-history, *n* = 4, 7, respectively, *p* = 0.0416) are shown. *p < 0.05, *t*-test.

We further analyzed the relationship between clinical histories. Febrile seizure is known as a major precipitating factor for the development of mesial temporal lobe epilepsy (Berg, 2008). Eight of the 12 patients had a history of febrile seizures (Supplementary Table 3). Phosphorylation at GluA1 Ser831 (GluA1pS831) was significantly increased in hippocampal lesions of patients with a previous history of febrile seizures (Figure 3). The age at surgery and onset (Supplementary Figures 4A,B, respectively), disease duration (Supplementary Figure 4C), storage periods of specimens (Supplementary Figure 4D), or FSIQ (Supplementary Figure 4E) and distribution of patients with or without febrile convulsions are shown.

## Discussion

Uncontrollable, sudden augmentation of hypersynchronous excitatory synaptic transmission in a subset of neurons in the cerebrum, along with impairment of proper suppression of recruiting hyperexcitability, has been hypothesized to be involved in the occurrence of focal epileptic seizures (De Curtis and Gnatkovsky, 2009; Gafurov and Bausch, 2013; Devinsky et al., 2018). To clarify the detailed molecular mechanism that forms the basis of the pathology of epilepsy, solid neurochemical methodologies were employed to detect posttranslational synaptic protein modifications and quantify their expression levels in patient brain tissues. Although analysis of biochemical extraction from homogenized brain tissue essentially evaluates a mixture of synaptic, extra-synaptic, and intracellular pools of synaptic proteins, Western blotting using specific antibodies enables us to quantify and compare total protein amounts as well as phosphorylation-regulated synaptic expression of the proteins examined. These methods have been established in our previous studies using the cerebral cortex, hippocampus, or amygdala of rodent brains (Itoh et al., 2018; Oota-Ishigaki et al., 2022). As demonstrated in the current study, the same approaches can be applied to recognize changes in protein modification and expression in the human brain. Experimentally, the material hardness of brain tissues differs considerably between rodents and humans. Tissue blocks from dissected frozen patient brain slices needed to be rapidly pulverized using a Multi-beads Shocker tissue homogenizer before treatment with detergent-containing lysis buffer as described in the “Materials and Methods” section. Then, the previously established protocol in rodent models could be applied for molecular analysis. The results revealed several characteristic features of hippocampal sclerosis, including disturbances in AMPA receptor phosphorylation in epileptogenic lesions.

**Figure 4:**
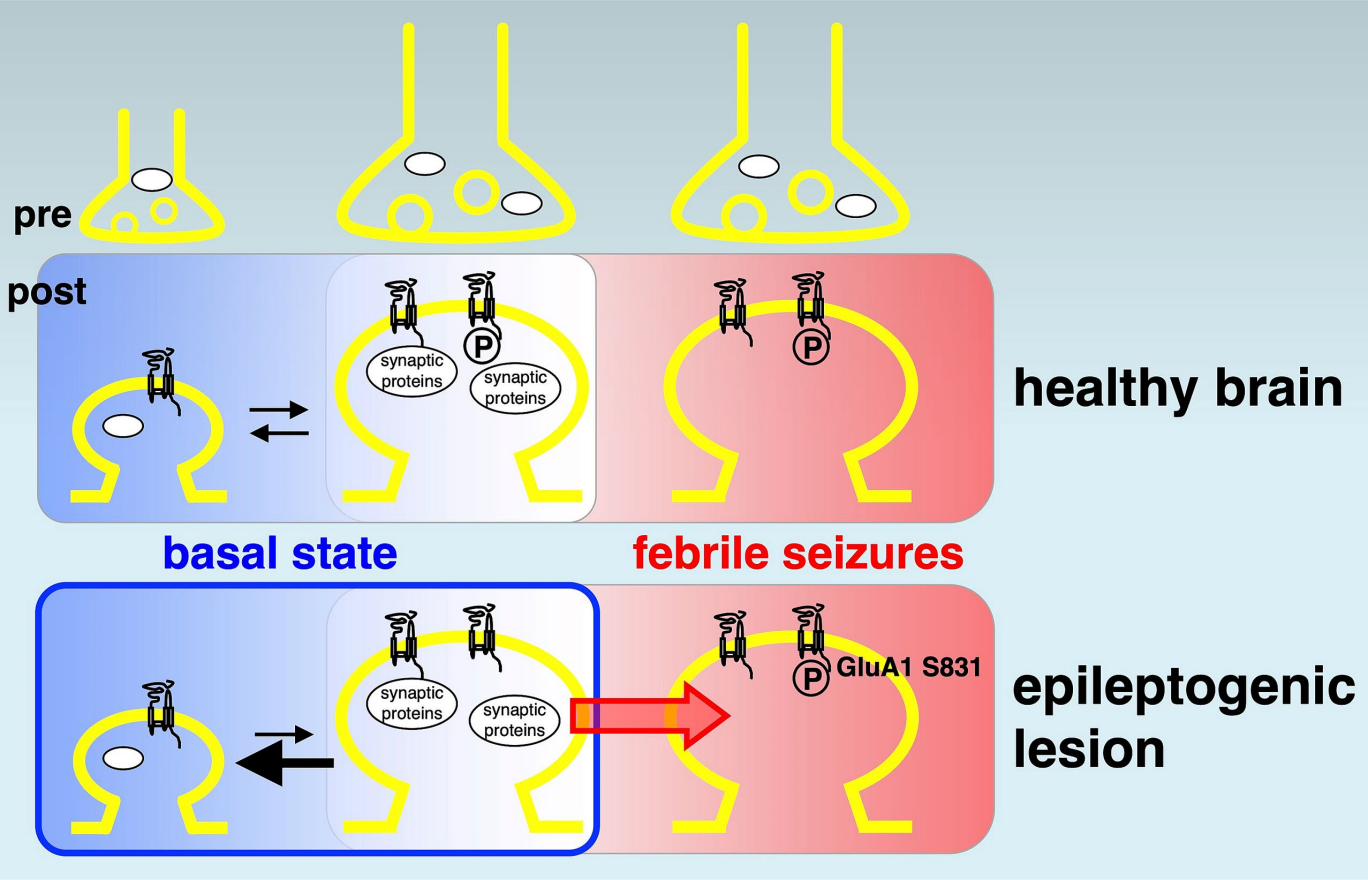
Potential model of reduced synaptic proteins and AMPA receptor phosphorylations in epileptogenesis. A model of disturbed synaptic proteins in epileptogenic lesions. In the basal state (left rounded rectangles, shown in blue background), many synaptic proteins and AMPA receptor phosphorylations (circled capital letter P) variously control up (towards right)- and down (towards left)-bidirectional regulation of physiological neural activity in the healthy brain (top). Chronically reduced synaptic protein expression (bottom, left, bold black arrow pointing left) induces the basal hypoactivity and hypometabolism in epileptogenic lesions. It may function as a fundamental mechanism of epileptogenesis (blue colored rounded rectangle). A history of febrile seizures (right rounded rectangles, shown in red background) is associated with increased AMPA receptor phosphorylation at GluA1 Ser831, which facilitates a transition towards reduced thresholds of hyperexcitation (red arrow) and correlates with the enhancement of local excitatory synaptic strength in the epileptogenic lesion (bottom, right).

The normal hippocampus contains a higher density of excitatory neurons compared to the anterior temporal neocortex, which would consequently imply a greater presence of GluRs and synaptic proteins in the healthy hippocampus. Corresponding to the overall hypoactivity and hypometabolism in the epileptogenic lesion of patients (von Oertzen, 2018), most patients showed fewer GluRs and pre- and postsynaptic proteins in the hippocampal epileptogenic lesion than those in the less epileptogenic control of the anterior temporal neocortex from the same patient (Table 1). A chronic decrease in synaptic protein expression generally reduces synaptic functions in epileptogenic lesions (Figure 4). The ubiquitously expressed protein GAPDH exhibited little difference between the control and epileptogenic lesions (Table 1, Figure 1, and Supplementary Figure 2). This indicates that total cell numbers, including neurons and glial cells, as well as protein amounts in the epileptogenic lesion, were maintained at the same level as in the control region, whereas a broad reduction of synaptic proteins occurred before the experiment due to repeated epileptic insults. Previous studies have revealed that mesial temporal lobe epilepsy is associated with neuronal loss and glial proliferation (gliosis) (Bote et al., 2008; Eid et al., 2013; Eid et al., 2008), which suggests the possibility that neuronal loss could influence the decreased GluRs and synaptic protein expression. The significantly decreased expression of some synaptic proteins, including the AMPA receptor GluA2 subunit, was also assessed by the average rate of change in tissue from the epileptogenic lesion (Figure 1 and Supplementary Figure 2). Adult mammalian hippocampus expresses mainly GluA1, GluA2, and GluA3, which form functional AMPA receptors consisting of GluA1/GluA2 or GluA2/GluA3 heterotetramers (Kessels and Malinow, 2009; Hayashi, 2021). Especially, GluA2 is the primary determinant during endocytosis of AMPA receptors from excitatory synapses (Anggono and Huganir, 2012). GluA4, which showed no apparent difference in the epileptic lesion, mainly expresses during the early postnatal development of the hippocampal circuit and has little effect on matured cerebral function. The NMDA receptor essential subunit GluN1 and its regulatory subunits GluN2A and GluN2B also showed significantly decreased expression in the epileptic lesion (Figure 1 and Supplementary Figure 2). Chronic reduction of AMPA receptors and NMDA receptors on the postsynaptic membrane leads to weakening of basal excitatory synaptic strength. Moreover, a decrease in some pre- and postsynaptic protein amounts disturbs the proper control of excitatory synaptic functions in epileptogenic lesions. Much literature has reported that agonists of AMPA or NMDA receptors can experimentally cause seizures in animals and humans. Moreover, some ASMs aiming at the inhibition of GluRs have been used clinically. A chronic decrease in GluRs and some synaptic protein expression seems contradictory to the knowledge. We consider that the most probable mechanism explaining these results is excessive local concentration of excitatory neurons and/or excitatory connections. These local abnormalities may occur at least partly in the microscopic regions of hippocampal lesions. In contrast, our biochemical analysis showed that GluRs and some synaptic proteins were decreased as an overall average in total tissue lysate. As a result of assumed local neuronal network dysfunction, GluRs agonist stimulation to overcrowded excitatory neurons can easily induce hyperexcitability as well as excessive synchronization of excitatory neurons. Variations were also observed among patients, and several patients showed increased GluRs and synaptic proteins in the epileptic lesion. In that case, a chronic increase of these proteins may spontaneously provoke an imbalance of total neuronal excitability.

Furthermore, posttranslational protein phosphorylations can lead to epilepsy-related alterations. Several studies have shown that GluA1pS831 generally increases AMPA receptor surface expression, which aligns with the physiological augmentation of mammalian excitatory synapses, such as long-term potentiation in learning and memory. In addition, GluA1pS845 acts synergistically for maximal synaptic excitation (Anggono and Huganir, 2012; Diering and Huganir, 2018). Thus, almost unchanged GluA1pS831 and significantly decreased GluA1pS845 were indisposed to energize neuronal activities in the entire epileptogenic lesion of patients (Figure 2 and Supplementary Figure 3). The overall weakening of excitatory synapses in epileptogenic lesions may reflect a total functional decline, including a decrease in the spiking frequency of neuronal networks and the duration of firing (Attwell and Laughlin, 2001; Tankus, 2016). Unlike GluA1 phosphorylation, both GluA2 phosphorylation sites at Ser880 and Tyr876 induce internalization of synaptic AMPA receptors through the exchange of GluA2-binding proteins from surface AMPA receptor-sustaining GRIP1 to AMPA receptor endocytosis-associated PICK1, resulting in the weakening of excitatory synaptic strength (Diering and Huganir, 2018). Reduced GluA2 phosphorylation in the epileptogenic lesion of most patients (Table 1E) and the comparative total decrease of GluA2pS880 (Figure 2 and Supplementary Figure 3) should reflect a deficiency of hyperexcitation-suppressing AMPA receptor internalization from the synaptic surface. Reduction of GluA2 phosphorylation may not decrease excessive synaptic activity in epileptogenic lesions once a trigger for hyperexcitability and hypersynchronization has formed in some overexcited groups of neurons. In contrast, the average rate of GluA2pY876 changes did not show any significant difference between the epileptogenic lesion and control regions. GluA2pY876 is related to homeostatic synaptic scaling, which involves the total tuning of whole neuronal networks (Turrigiano, 2008; Turrigiano, 2017). The mechanism of suppression of excitatory synapses by either GluA2pS880 or GluA2pY876 may function in different time windows.

A febrile convulsion-dependent increase in GluA1pS831 levels was observed in the brains of patients (Figure 3). A history of febrile seizures was associated with upregulation of basal phosphorylation at GluA1 Ser831 in six out of eight patient control regions and six out of seven patient epileptic lesions, which may correlate with the enhancement of basal excitatory synaptic strength in patients, reducing the threshold of hyperexcitability (Rakhade et al., 2012). A recent analysis using cross-sectional PET imaging with a radiotracer for AMPA receptors, [^11^C]K-2, revealed that epileptic brain functions can be regulated by the enhanced trafficking of AMPA receptor due to Hebbian plasticity with increased simultaneous neuronal firing and compensational downregulation of cell-surface AMPA receptors by the homeostatic synaptic scaling in patients with epilepsy (Eiro et al., 2023). These macroscopic observations are consistent with the molecular changes shown here, regarding the febrile convulsion-dependent upregulation of basal phosphorylation at GluA1 Ser831 and the chronic decreases in GluRs and synaptic protein expression, respectively.

## Conclusion

This study revealed molecular disturbance of synaptic proteins in the epileptogenic lesion of patients, which is considered at least part of the basis for drug-resistant focal epilepsy. The reduced pre- and postsynaptic protein expression, including excitatory glutamate receptors, indicates an overall weakening of synapses in the chronic condition. This may explain clinically observed basal hypoactivity and hypometabolism in epileptogenic lesions and may function as a fundamental mechanism of epileptogenesis. Moreover, a history of febrile seizures was associated with increased AMPA receptor phosphorylation, which correlates with the enhancement of excitatory synaptic strength and reduced thresholds of hyperexcitation.

## Data Availability

The original contributions presented in the study are included in the article/Supplementary material, further inquiries can be directed to the corresponding author.

## Footnotes

^1^ https://ncbiobank.org/en/home.php

## Supplementary material

SUPPLEMENTARY TABLE 1 Correlation of protein amounts with characteristics of patients. Correlation coefficients of AMPA receptor subunits GluA1–4 (A), NMDA receptor subunits GluN1, GluN2A, and GluN2B (B), postsynaptic proteins (C), presynaptic proteins (D), and GAPDH protein (E) with age at surgery, age of onset, disease duration, storage period of the specimen, and pre-operative full-scale intelligence quotient (FSIQ) are shown.

SUPPLEMENTARY TABLE 2 Correlation of protein modification with characteristics of patients. Correlation coefficients of AMPA receptor phosphorylation with age at surgery, age of onset, disease duration, storage period of the specimen, and pre-operative full-scale intelligence quotient (FSIQ) are shown.

SUPPLEMENTARY TABLE 3 Clinical information on individual patients. Patient characteristics in this study. Sex: male (M) or female (F), side of surgery: left (L) or right (R) hemisphere, age at surgery (years), age of onset (years), disease duration (years), past medical history: acute encephalopathy with biphasic seizure and late reduced diffusion (AESD), family history, full-scale intelligence quotient (FSIQ), are shown.

SUPPLEMENTARY FIGURE 1 Epilepsy-induced changes of synaptic protein expression in each patient. Variability in pre- and post-synaptic protein expression patterns among 12 patients (Pt1-Pt12) between control (adjusted to 100%) and the epileptogenic lesion in each patient are shown. **(A)** Comparison of protein amounts of AMPA receptor subunits: GluA1 protein amounts (white bars), GluA2 protein amounts (black bars), GluA3 protein amounts (gray bars), and GluA4 protein amounts (striped bars). **(B)** Comparison of protein amounts of NMDA receptor subunits: GluN1 protein amounts (white bars), GluN2A protein amounts (gray bars), and GluN2B protein amounts (black bars). **(C)** Comparison of presynaptic protein amounts: Synaptotagmin-1 protein amounts (white bars), Synaptophysin protein amounts (gray bars), and Synapsin-1 protein amounts (black bars). **(D)** Comparison of postsynaptic protein amounts: PSD95 protein amounts (white bars), SAP102 protein amounts (dotted bars), GRIP1 protein amounts (gray bars), PICK1 protein amounts (striped bars), and SynGAP protein amounts (black bars). Protein expression levels in the epileptogenic lesions were normalized to control, indicated by a dotted line (100%). Bar border colors are marked as follows: upregulated in the epileptogenic lesion (red), downregulated in the epileptogenic lesion (blue), subtle differences within 100 ± 5% in the epileptogenic lesion (black), compared to control. X indicates unevaluable data because signals were quantitatively undetectable in control. The original immunoblots are shown in Supplementary Source Data.

SUPPLEMENTARY FIGURE 2 Epilepsy-induced changes of synaptic proteins expression. Comparison of protein amounts between control (white bars, defined as 100%) and epileptogenic lesion (gray bars) lysates. **(A)** GluR protein expression: AMPA receptor subunits, GluA1 (217.7 ± 132.6%, compared to control, *n* = 11, respectively; *p* = 0.2662), GluA2 (169.57 ± 91.02%, compared to control, *n* = 12, respectively; *p* = 0.1461), GluA3 (726.7 ± 540.2%, compared to control, *n* = 12, respectively; *p* = 0.1461), and GluA4 (119.14 ± 63.94%, compared to control, *n* = 6, respectively; *p* = 0.2987); NMDA receptor subunits, GluN1 (29.69 ± 15.28%, compared to control, *n* = 6, respectively; *p* = 0.0022), GluN2A (75.63 ± 54.58%, compared to control, *n* = 7, respectively; *p* = 0.1474), and GluN2B (20.41 ± 10.22%, compared to control, *n* = 10, respectively; *p* < 0.0001). **(B)** Postsynaptic proteins: PSD-95 (342.8 ± 214.3%, compared to control, *n* = 12, respectively; *p* = 0.2813), SAP102 (841.8 ± 778.5%, compared to control, *n* = 10, respectively; *p* = 0.1138), GRIP1 (484.0 ± 326.1%, compared to control, *n* = 12, respectively; *p* > 0.9999), PICK1 (282.1 ± 231.3%, compared to control, *n* = 12, respectively; *p* = 0.4477), and SynGAP (37.97 ± 23.77%, compared to control, *n* = 9, respectively; *p* = 0.0361). **(C)** Presynaptic proteins: Synaptotagmin-1 (746.3 ± 601.8%, compared to control, *n* = 10, respectively; *p* = 0.4354), Synaptophysin (67.76 ± 18.65%, compared to control, *n* = 12, respectively; *p* = 0.0255), and Synapsin-1 (101.17 ± 20.32%, compared to control, *n* = 12, respectively; *p* > 0.9999). **(D)** Ubiquitous expressing protein, GAPDH (173.22 ± 80.25%, compared to control, *n* = 12, respectively; *p* > 0.9999). **p* < 0.05, ***p* < 0.01, *****p* < 0.0001, Mann-Whitney U test. **(E)** Variability of protein expressions in control anterior temporal neocortex. Distribution of protein amounts, adjusted to average of each protein. AMPA receptor subunits, GluA1 (100 ± 17.90%, *n* = 11), GluA2 (100 ± 18.90%, *n* = 12), GluA3 (100 ± 18.26%, *n* = 12), and GluA4 (100 ± 55.45%, *n* = 6); NMDA receptor subunits, GluN1 (100 ± 31.62%, *n* = 6), GluN2A (100 ± 20.59%, *n* = 7), and GluN2B (100 ± 24.39%, *n* = 10); Postsynaptic proteins, PSD-95 (100 ± 22.96%, *n* = 12), SAP102 (100 ± 16.37%, *n* = 10), GRIP1 (100 ± 21.83%, *n* = 12), PICK1 (100 ± 43.20%, *n* = 12), and SynGAP (100 ± 32.07%, *n* = 9); Presynaptic proteins, Synaptotagmin-1 (100 ± 27.38%, *n* = 10), Synaptophysin (100 ± 19.61%, *n* = 12), and Synapsin-1 (100 ± 12.01%, *n* = 12). GAPDH in control (white bar, 100 ± 23.51%, *n* = 12) and hippocampal epileptogenic lesions (gray bar, 100 ± 29.69%, *n* = 12) are shown as a comparison.

SUPPLEMENTARY FIGURE 3 Epilepsy-induced changes of GluR protein phosphorylation. Ratios of GluA1 and GluA2 protein phosphorylation in individual specimens prepared from patient anterior temporal neocortex (odd-numbered samples) or hippocampal lesions (even-numbered samples), which were normalized to the amount of GluA protein in the same lysates [left panels, in arbitrary units (a.u.)]. Odd and even sample pairs indicate control and epileptogenic lesions in each patient (Pt1-Pt12). Bars are marked as follows: upregulated in the epileptogenic lesion (red), downregulated in the epileptogenic lesion (blue), and subtle differences within 100 ± 5% in the epileptogenic lesion (white), compared to that of the control or unevaluable (yellow) because signals were quantitatively undetectable in control. **(A)** GluA1 phosphorylation at Ser831 (GluA1pS831) was normalized to the GluA1 protein amounts (GluA1pS831/GluA1, *n* = 10). **(B)** GluA1 phosphorylation at Ser845 (GluA1pS845) was normalized to GluA1 protein amounts (GluA1pS845/GluA1, *n* = 6). **(C)** GluA2 phosphorylation at Ser880 (GluA2pS880) was normalized to GluA2 protein amounts (GluA2pS880/GluA2, *n* = 6). **(D)** GluA2 phosphorylation at Tyr876 (GluA2pY876) was normalized to GluA2 protein amounts (GluA2pS876/GluA2, *n* = 6). The original immunoblots are shown in Supplementary Source Data. **(E)** Alteration of GluA1 and GluA2 protein phosphorylation in 12 patients (Pt1-Pt12). Ratio of GluA1 or GluA2 phosphorylation to each GluA protein amounts in the epileptogenic lesion was normalized to control, indicated by a dotted line (100%). GluA1pS831 (white bars), GluA1pS845 (white striped bars), GluA2pS880 (black bars), and GluA2pS876 (gray striped bars) are shown. Bar border colors are marked as follows: upregulated in the lesion (red), downregulated in the lesion (blue), subtle differences within 100 ± 5% in the lesion (black), compared to control. X indicates unevaluable data because signals were quantitatively undetectable in control.

SUPPLEMENTARY FIGURE 4 Classification of patients concerning febrile convulsion experiences. Distribution of patients according to each characteristic. Patients with or without previous febrile convulsions are shown: **(A)** Age at surgery (years, *p* = 0.0659). **(B)** Age of onset (years, *p* = 0.2039). **(C)** Disease duration (years, *p* = 0.2551). **(D)** Storage period of specimen (days, *p* = 0.8487). **(E)** Full-scale intelligence quotient (FSIQ, *p* = 0.8746). *t* test. Error bars represent s. d.

SUPPLEMENTARY FIGURE 5 Schematics of sample preparation. Serial 5 mm-thick brain slice sections have been registered in the NCNP biobank, which were surgically obtained from the hippocampal lesion (top, left) or temporal lobe tip (bottom, left) of individual patients. Every serial slice was alternately fixed (red) or rapidly frozen (blue) during a surgical operation. Fixed sections were followed by pathological diagnosis of sclerosis (right). Epileptogenic legions (top, grey oval) should be located side by side in the serial fixed and frozen sections. Then, a cracked small frozen tissue block (blue square) from the corresponding region in neighboring frozen hippocampal section (surrounded by dotted line) was used for biochemical experiments as the epileptogenic lesion. Healthier and less epileptogenic region was similarly prepared from the frozen temporal lobe tip section as control (bottom).

SUPPLEMENTARY SOURCE DATA Immunoblots of synaptic proteins expression and GluR protein phosphorylation in patients. A series of original western blots showing protein expression levels and GluR protein phosphorylation in specimens from the control anterior temporal neocortex (odd-numbered samples) and those from hippocampal epileptogenic lesions (even-numbered samples) of patients 1–6 (top) and patients 7–12 (bottom). Relative molecular masses of markers (M) are shown at the left.
