# Supplementary material for: Chronic reduction of synaptic proteins in the epileptogenic lesion of patients with hippocampal sclerosis
Source: Front Mol Neurosci. 2025 Jul 23;18:1635852. doi: 10.3389/fnmol.2025.1635852 (PMC12325362; doi:10.3389/fnmol.2025.1635852)
Supplement: Supplementary file 1 [file Data_Sheet_1.pdf]

A Correlation coefficients in AMPA receptor protein amounts (*r*)

	GluA1 (% in lesion, compared with control)	GluA1 in temporal lobe tip	GluA1 in hippocampus	GluA2 (% in lesion)	GluA2 in temporal lobe tip	GluA2 in hippocampus
age at surgery	-0.11	0.15	-0.32	-0.10	0.06	-0.33
age of onset	0.10	0.21	0.07	0.09	0.14	-0.0003
disease duration	-0.21	0.02	-0.43	-0.19	-0.03	-0.39
storage period	0.50	-0.50	-0.33	0.49	-0.47	-0.69
FSIQ	0.40	0.07	0.23	0.38	-0.14	-0.02

	GluA3 (% in lesion, compared with control)	GluA3 in temporal lobe tip	GluA3 in hippocampus	GluA4 (% in lesion, compared with control)	GluA4 in temporal lobe tip	GluA4 in hippocampus
age at surgery	-0.32	0.01	-0.28	0.13	-0.33	0.09
age of onset	-0.16	0.18	0.09	0.44	-0.35	0.33
disease duration	-0.27	-0.12	-0.40	-0.10	-0.13	-0.14
storage period	0.33	-0.77	-0.56	-0.61	-0.23	-0.55
FSIQ	-0.24	0.08	0.22	0.31	-0.23	0.27

B Correlation coefficients in NMDA receptor protein amounts (*r*)

	GluN1 (% in lesion, compared with control)	GluN1 in temporal lobe tip	GluN1 in hippocampus	GluN2A (% in lesion, compared with control)	GluN2A in temporal lobe tip	GluN2A in hippocampus	GluN2B (% in lesion, compared with control)	GluN2B in temporal lobe tip	GluN2B in hippocampus
age at surgery	-0.63	0.04	-0.25	-0.35	0.10	-0.34	-0.39	-0.01	-0.41
age of onset	-0.68	0.54	0.14	-0.48	0.46	-0.03	-0.25	0.17	-0.21
disease duration	-0.17	-0.35	-0.40	0.05	-0.22	-0.38	-0.30	-0.13	-0.33
storage period	-0.45	-0.51	-0.42	-0.30	-0.31	-0.45	-0.57	-0.28	-0.57
FSIQ	-0.39	0.15	0.06	-0.16	-0.08	-0.0002	-0.02	-0.13	-0.12

C Correlation coefficients in postsynaptic protein amounts (*r*)

	PSD95 (% in lesion, compared with control)	PSD95 in temporal lobe tip	PSD95 in hippocampus	SAP102 (% in lesion, compared with control)	SAP102 in temporal lobe tip	SAP102 in hippocampus	GRIP1 (% in lesion, compared with control)	GRIP1 in temporal lobe tip	GRIP1 in hippocampus
age at surgery	-0.10	-0.02	-0.49	0.38	0.13	0.31	0.44	0.02	-0.40
age of onset	0.11	-0.19	-0.25	0.34	-0.07	0.03	0.04	0.16	0.07
disease duration	-0.19	0.11	-0.40	0.20	0.20	0.35	0.49	-0.09	-0.53
storage period	0.50	-0.02	-0.08	-0.29	0.63	0.45	0.48	-0.49	-0.49
FSIQ	0.39	-0.30	-0.43	0.43	-0.21	0.26	0.09	0.14	0.05

	PICK1 (% in lesion, compared with control)	PICK1 in temporal lobe tip	PICK1 in hippocampus	SynGAP (% in lesion, compared with control)	SynGAP in temporal lobe tip	SynGAP in hippocampus
age at surgery	-0.36	0.38	-0.08	-0.59	0.27	-0.44
age of onset	-0.21	0.14	-0.17	-0.42	0.24	-0.36
disease duration	-0.27	0.36	0.04	-0.35	0.14	-0.26
storage period	0.21	0.63	0.66	-0.44	-0.12	-0.51
FSIQ	-0.35	0.41	0.17	-0.20	0.07	-0.15

D Correlation coefficients in presynaptic protein amounts (*r*)

	Synaptotagmin-1 (% in lesion, compared with control)	Synaptotagmin-1 in temporal lobe tip	Synaptotagmin-1 in hippocampus	Synaptophysin (% in lesion, compared with control)	Synaptophysin in temporal lobe tip	Synaptophysin in hippocampus	Synapsin-1 (% in lesion, compared with control)	Synapsin-1 in temporal lobe tip	Synapsin-1 in hippocampus
age at surgery	-0.17	-0.21	-0.26	-0.29	0.16	0.12	-0.01	0.30	0.14
age of onset	-0.40	0.46	-0.41	0.12	0.30	0.50	-0.18	0.64	0.18
disease duration	0.12	-0.59	-0.005	-0.43	-0.04	-0.23	0.11	-0.12	0.04
storage period	0.11	-0.80	-0.01	0.25	-0.59	-0.72	0.49	-0.17	0.42
FSIQ	-0.18	-0.05	-0.41	0.31	0.05	0.32	-0.35	0.30	-0.13

E **Correlation coefficients in GAPDH protein amounts (*r*)**

	GAPDH (% in lesion, compared with control)	GAPDH in temporal lobe tip	GAPDH in hippocampus
age at surgery	0.35	0.15	0.02
age of onset	-0.003	0.46	0.46
disease duration	0.42	-0.17	-0.32
storage period	0.38	-0.38	-0.54
FSIQ	0.04	0.04	0.28

Correlation coefficients in phosphorylation (*r*)

	GluA1pS831/GluA1 (% in lesion)	GluA1pS831/GluA1 in temporal lobe tip	GluA1pS831/GluA1 in hippocampus	GluA1pS845/GluA1 (% in lesion)	GluA1pS845/GluA1 in temporal lobe tip	GluA1pS845/GluA1 in hippocampus
age at surgery	-0.02	-0.23	-0.24	0.27	0.46	-0.11
age of onset	-0.23	-0.13	-0.17	0.77	0.25	-0.17
disease duration	0.17	-0.17	-0.16	-0.32	0.36	-0.001
storage period	0.31	-0.60	-0.22	-0.14	-0.09	-0.31
FSIQ	-0.57	-0.14	-0.34	0.37	0.34	-0.11
	GluA2pS880/GluA2 (% in lesion)	GluA2pS880/GluA2 in temporal lobe tip	GluA2pS880/GluA2 in hippocampus	GluA2pY876/GluA2 (% in lesion)	GluA2pY876/GluA2 in temporal lobe tip	GluA2pY876/GluA2 in hippocampus
age at surgery	0.13	0.23	0.26	-0.40	-0.12	-0.33
age of onset	0.27	0.39	0.61	-0.67	0.41	-0.25
disease duration	-0.13	-0.01	-0.14	0.18	-0.44	-0.21
storage period	0.33	-0.51	-0.28	-0.39	-0.69	-0.54
FSIQ	0.42	0.33	0.37	-0.30	-0.05	-0.01

patient	sex	side of surgery	age at surgery (y)	age of onset (y)	disease duration (y)	past medical history	family history	FSIQ
1	M	L	35	9	26	febrile seizure	no	53
2	F	L	56	14	42	febrile seizure	no	75
3	M	L	32	18	14	no	no	99
4	F	R	18	8	10	febrile seizure	no	58
5	F	R	51	10	41	febrile seizure	no	90
6	M	L	21	3.5	17.5	no	no	68
7	M	R	46	31	15	febrile seizure	febrile seizure, epilepsy	91
8	F	R	28	9	19	no	no	69
9	F	L	52	24	28	febrile seizure	epilepsy	97
10	F	L	27	6	21	febrile seizure	febrile seizure	67
11	F	L	11	4	7	AESD	febrile seizure	67
12	M	L	28	25	3	febrile seizure	no	62

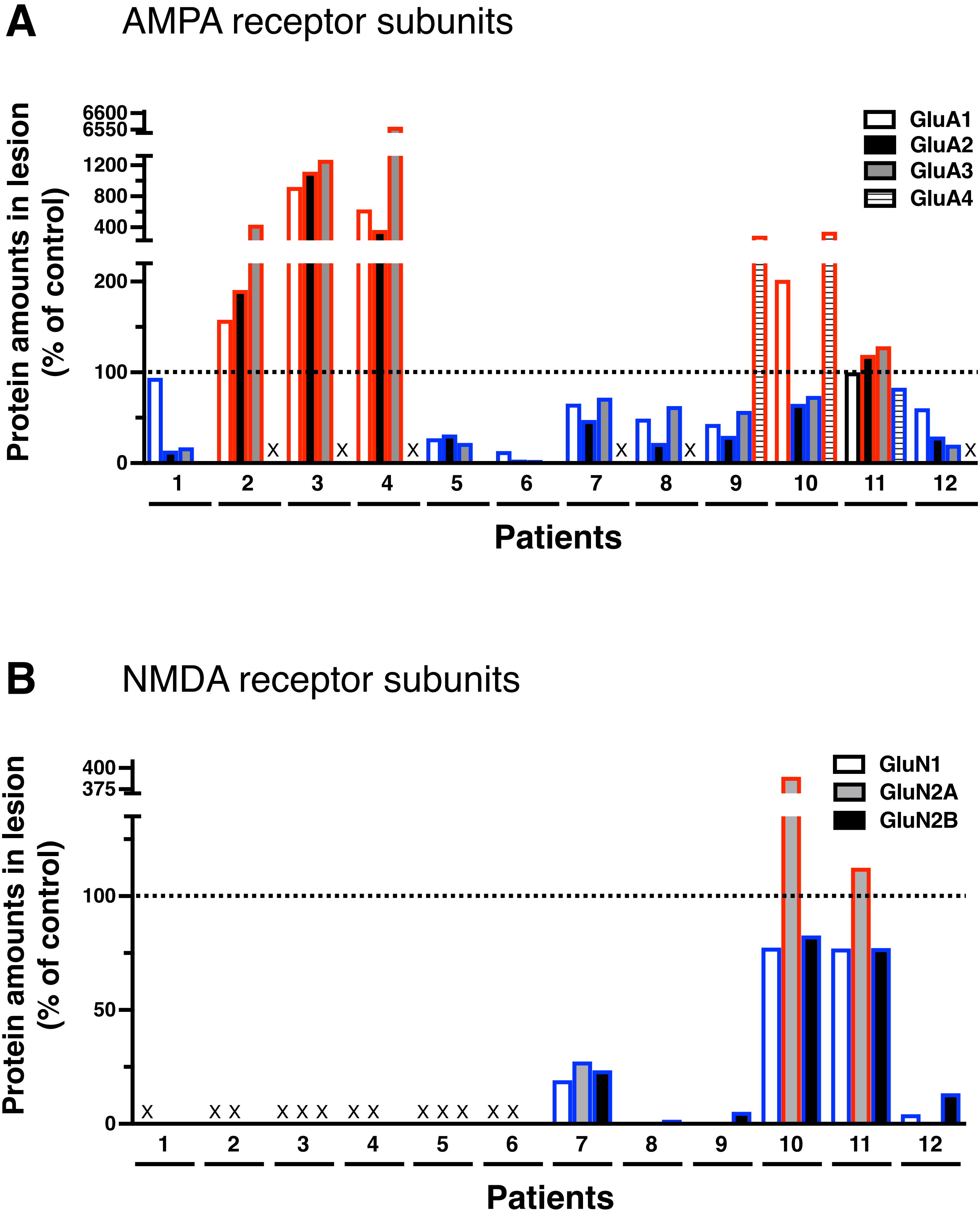

Oota-Ishigaki et al. Supple Fig. 1

C Presynaptic proteins

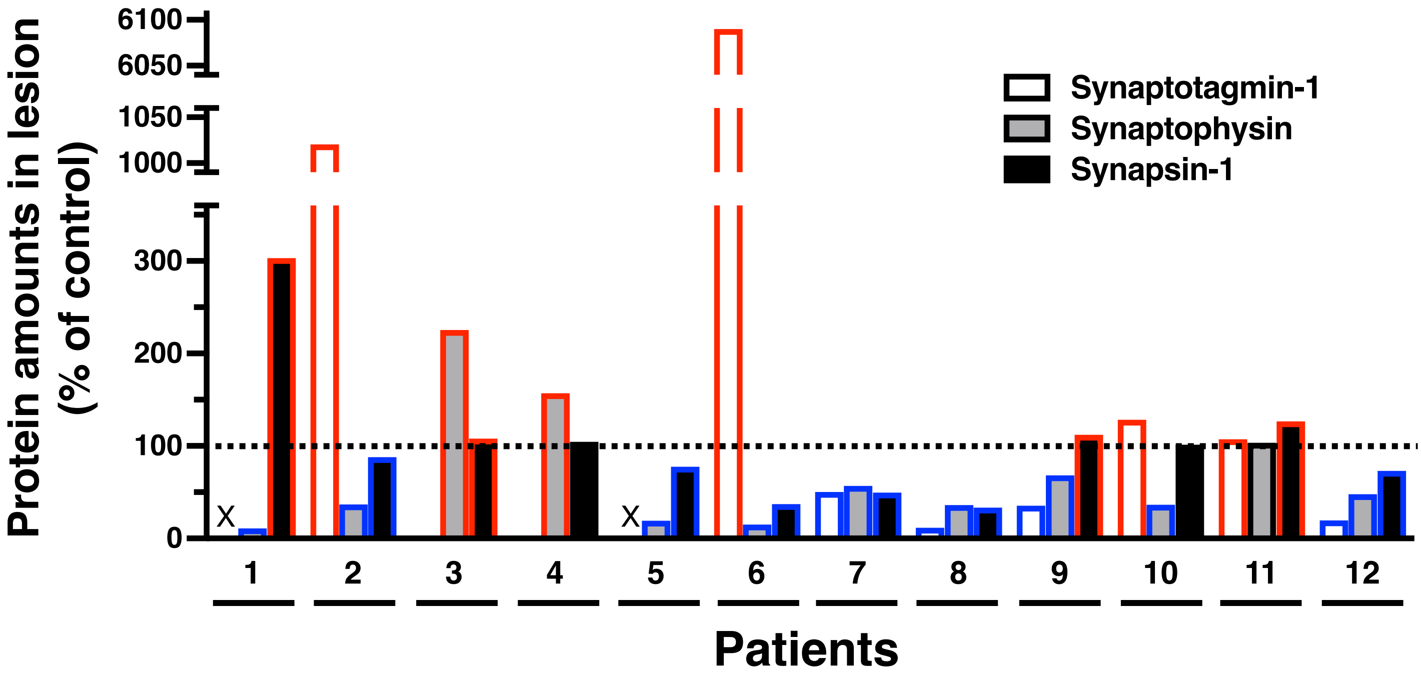

D Postsynaptic proteins

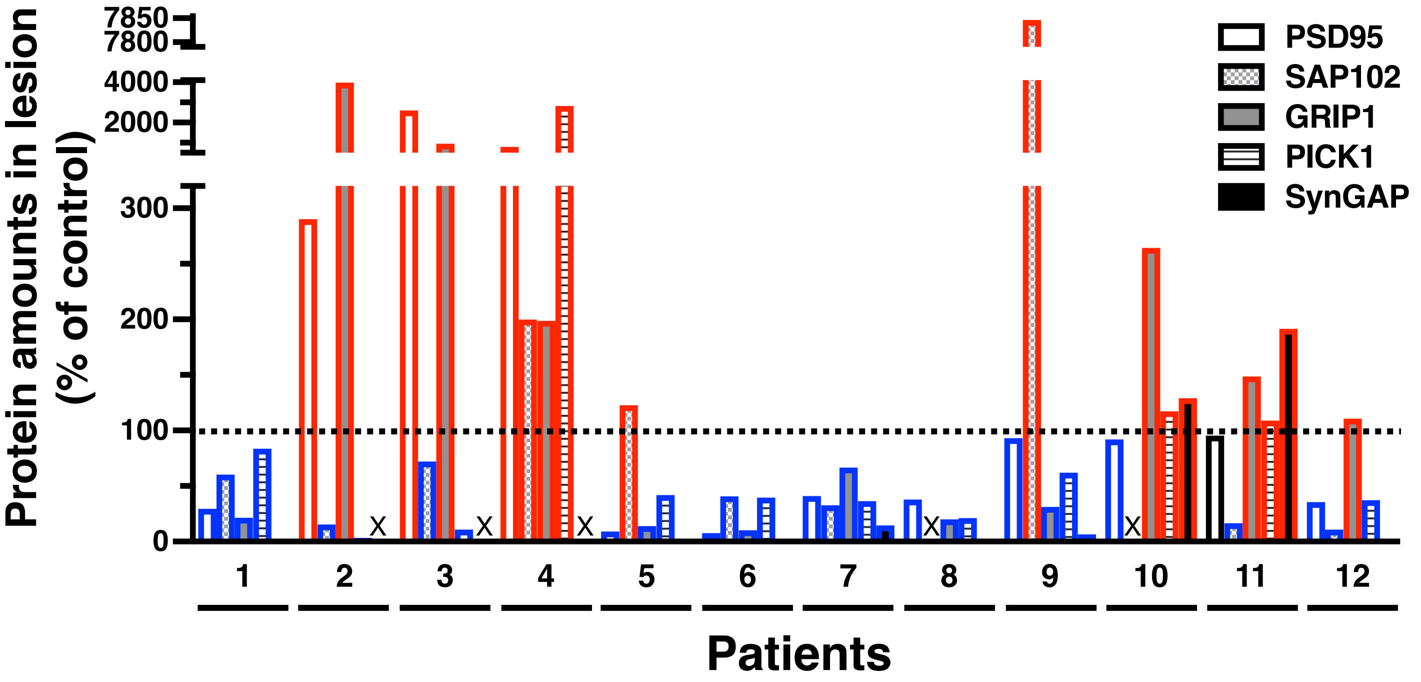

Oota-Ishigaki et al. Supple Fig. 1

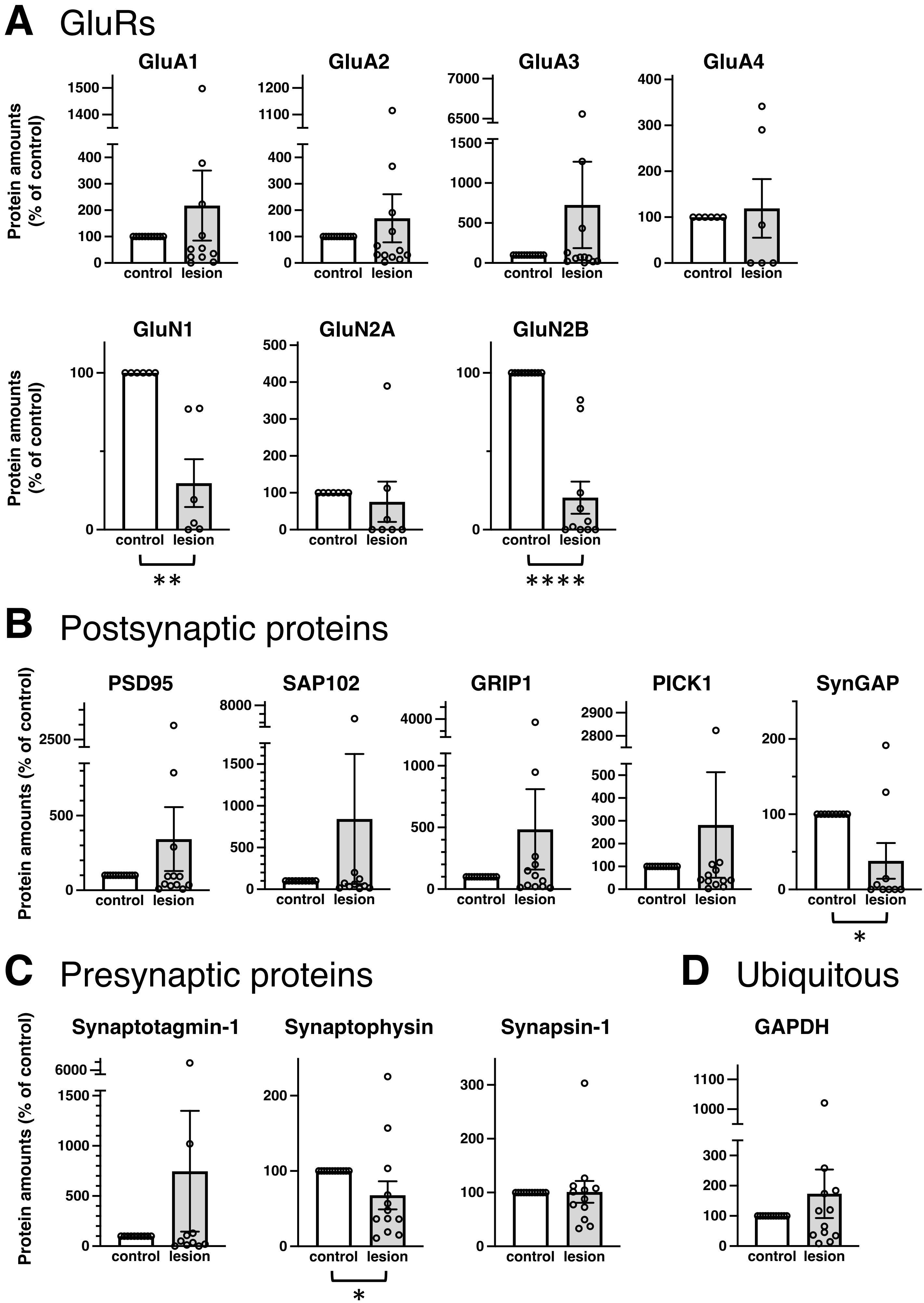

Oota-Ishigaki et al. Supple Fig. 2

E

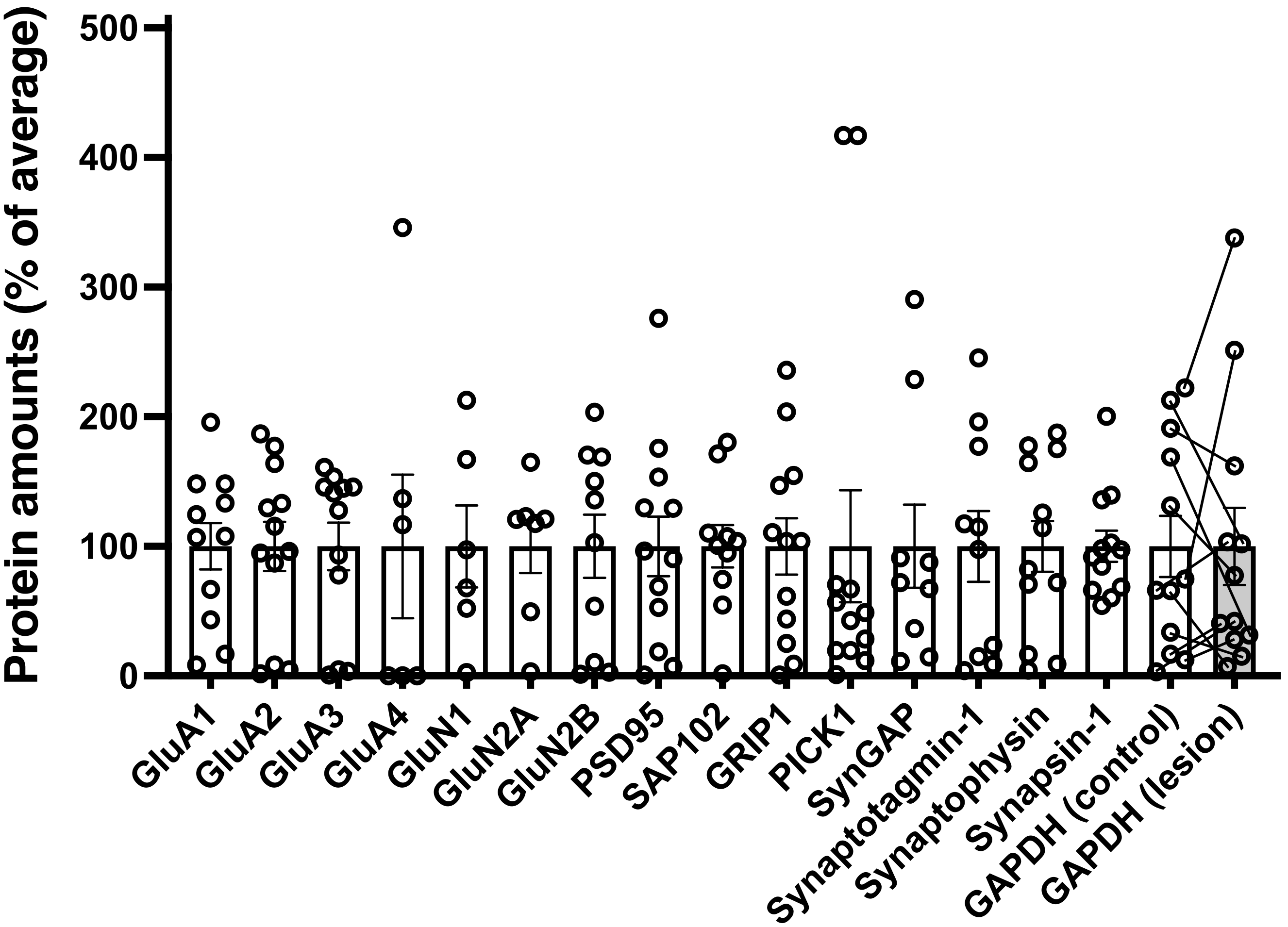

Oota-Ishigaki et al. Supple Fig. 2

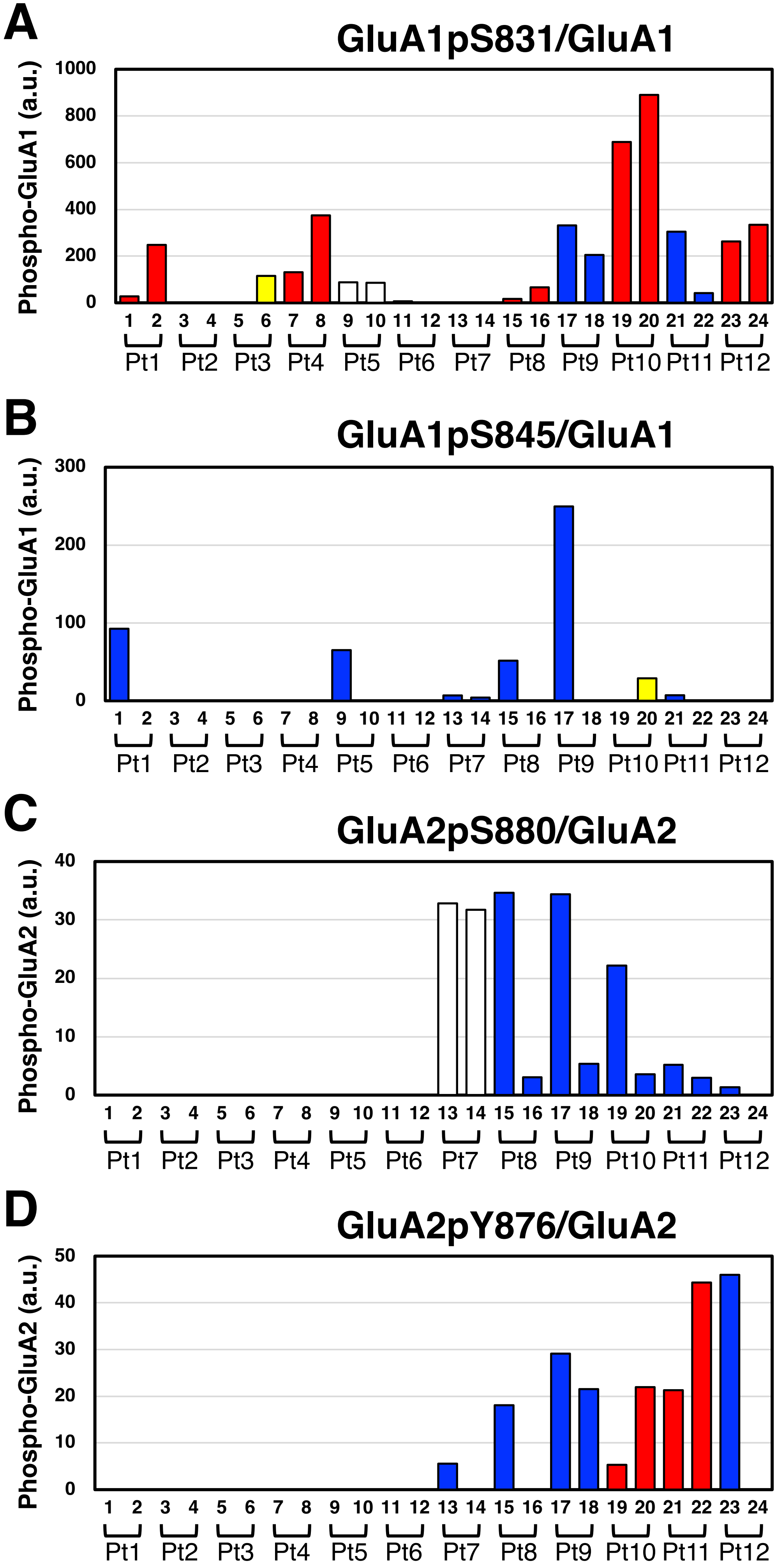

Oota-Ishigaki et al. Supple Fig. 3

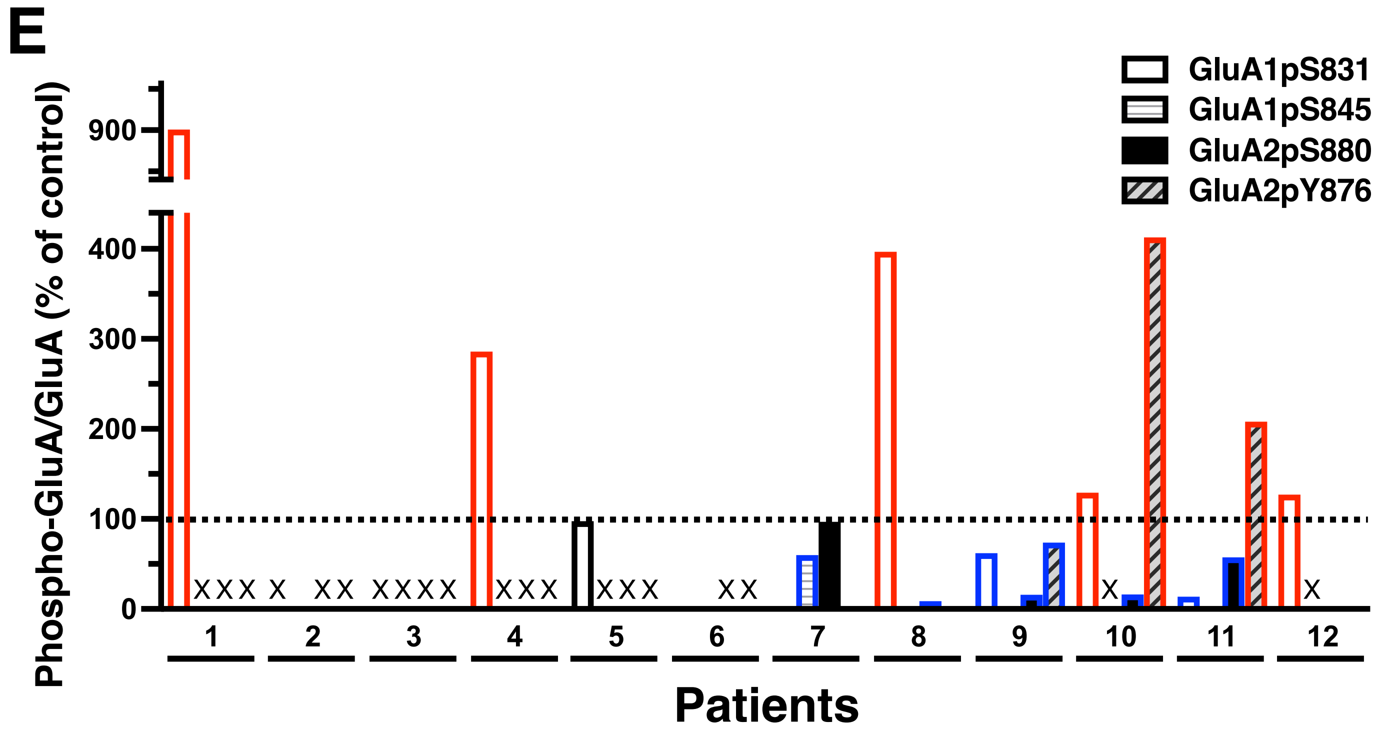
E
GluA1pS831
GluA1pS845
GluA2pS880
GluA2pY876
Phospho-GluA/GluA (% of control)
900
400
300
200
100
0
1
2
3
4
5
6
7
8
9
10
11
12
Patients

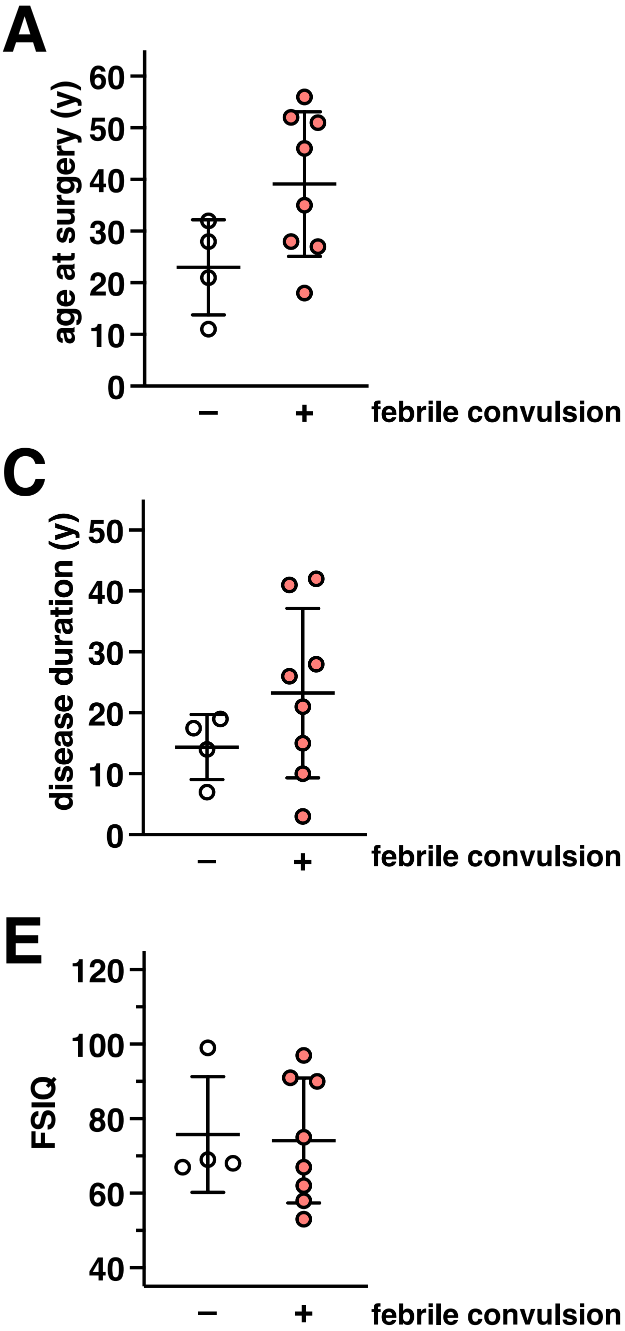

Oota-Ishigaki et al. Supple Fig. 4

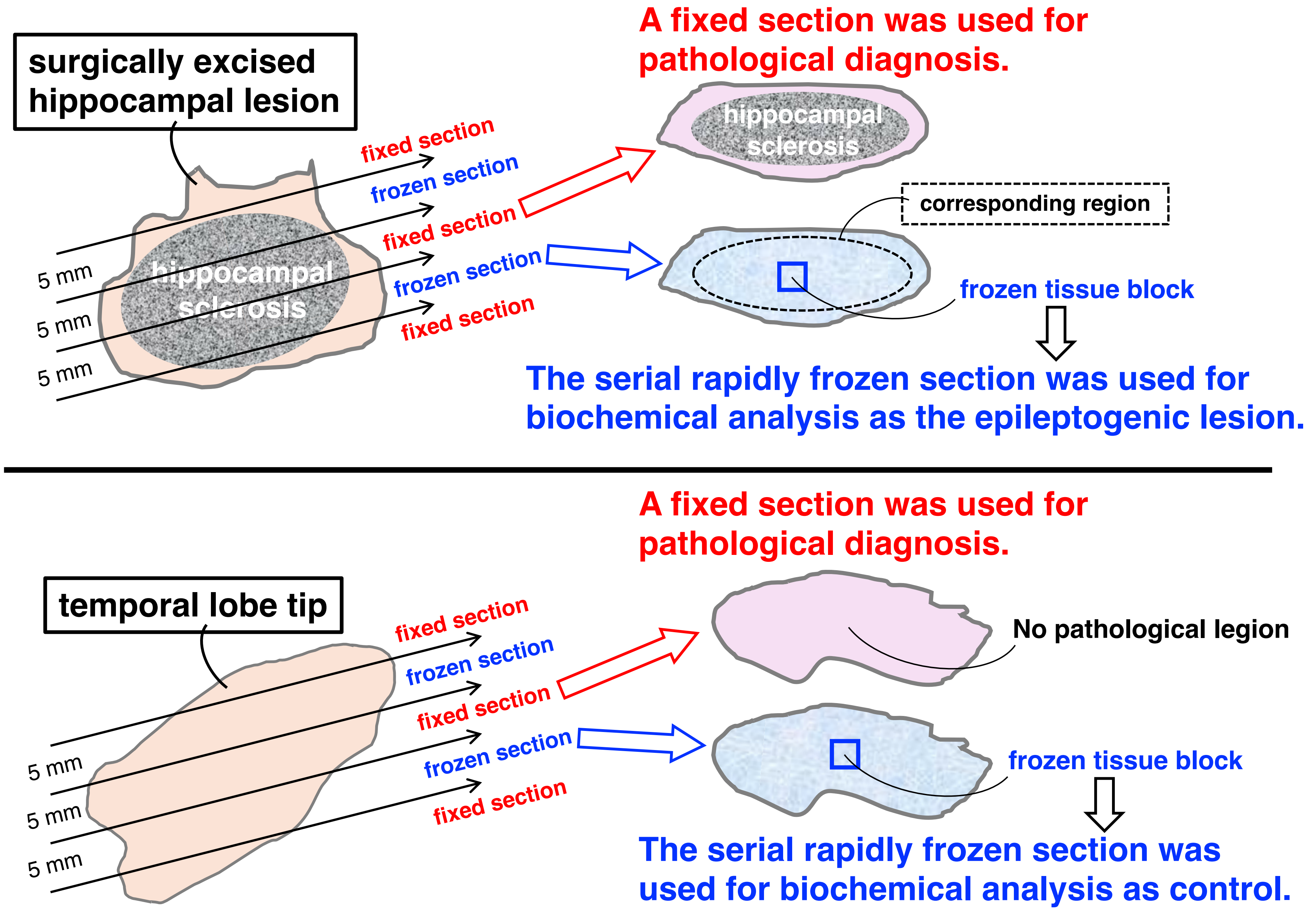

Oota-Ishigaki et al. Supple Fig. 5
