# Supplementary material for: Chronic reduction of synaptic proteins in the epileptogenic lesion of patients with hippocampal sclerosis
Source: Front Mol Neurosci. 2025 Jul 23;18:1635852. doi: 10.3389/fnmol.2025.1635852 (PMC12325362; doi:10.3389/fnmol.2025.1635852)
Supplement: Supplementary file 2 [file Data_Sheet_2.pdf]

Western blotting (top)						
	Patient 1	Patient 2	Patient 3	Patient 4	Patient 5	Patient 6
Specimen from control temporal lobe tip<br>Specimen from the hippocampal epileptogenic lesion	No. 1<br>No. 2	No. 3<br>No. 4	No. 5<br>No. 6	No. 7<br>No. 8	No. 9<br>No. 10	No. 11<br>No. 12

Western blotting (bottom)						
	Patient 7	Patient 8	Patient 9	Patient 10	Patient 11	Patient 12
Specimen from control temporal lobe tip<br>Specimen from the hippocampal epileptogenic lesion	No. 13<br>No. 14	No. 15<br>No. 16	No. 17<br>No. 18	No. 19<br>No. 20	No. 21<br>No. 22	No. 23<br>No. 24

GluA1

long exposure

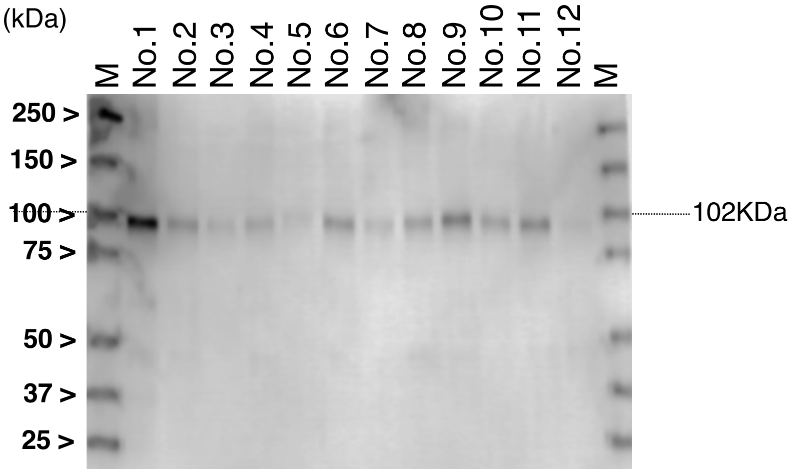

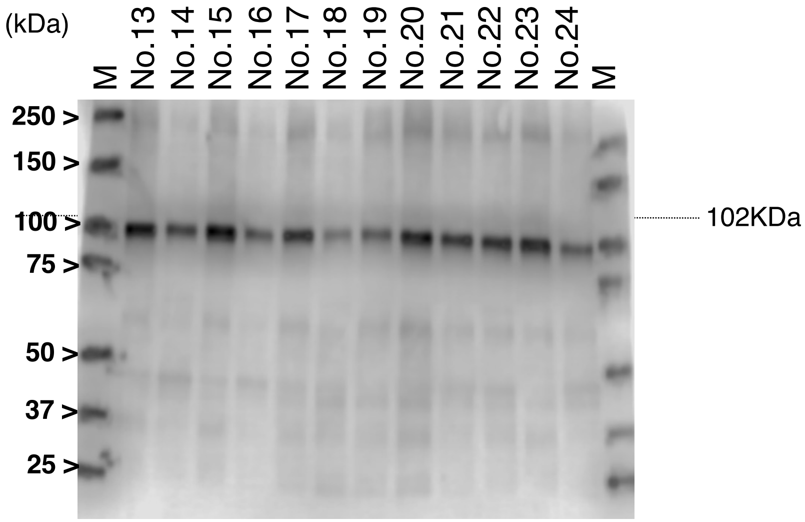

short exposure

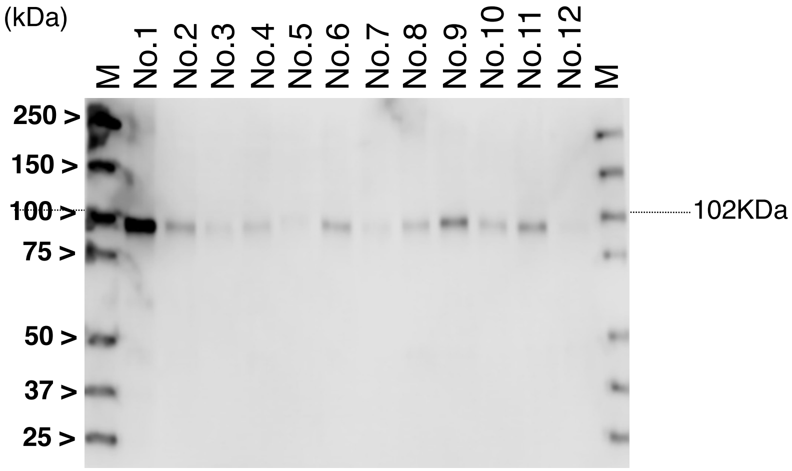

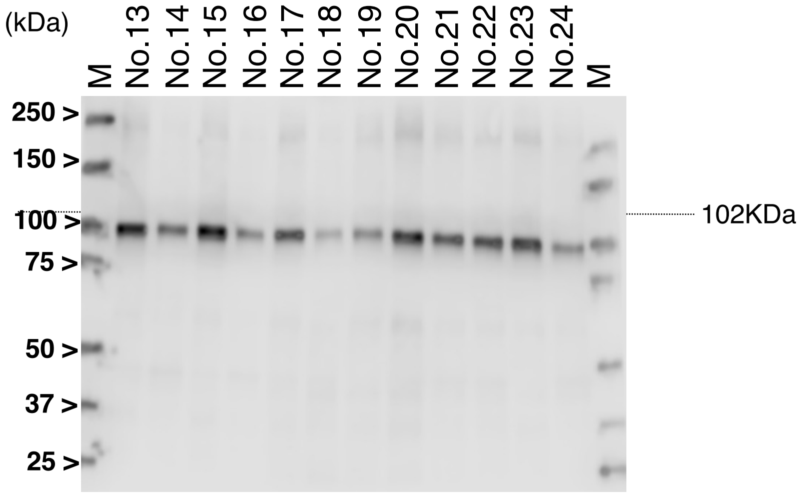

Oota-Ishigaki et al. Source Data

GluA2

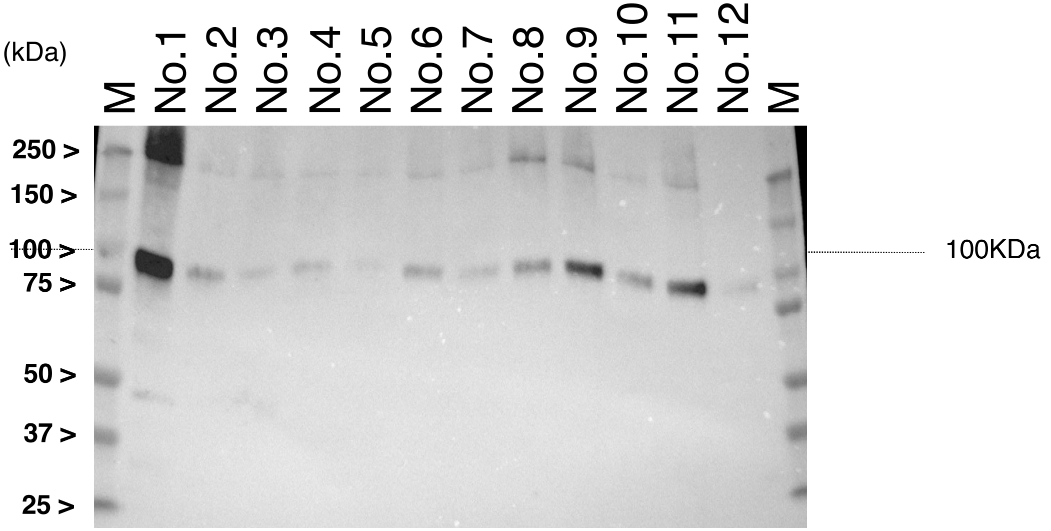

(kDa)
M
No.1
No.2
No.3
No.4
No.5
No.6
No.7
No.8
No.9
No.10
No.11
No.12
M
250 >
150 >
100 >
75 >
50 >
37 >
25 >
100KDa

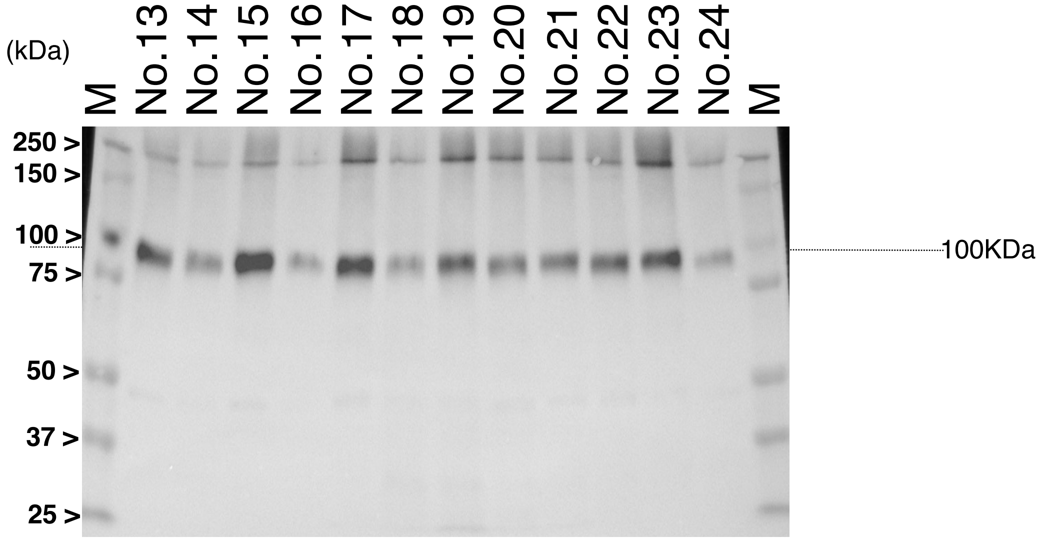

(kDa)
M
No.13
No.14
No.15
No.16
No.17
No.18
No.19
No.20
No.21
No.22
No.23
No.24
M
250 >
150 >
100 >
75 >
50 >
37 >
25 >
100KDa

GluA3

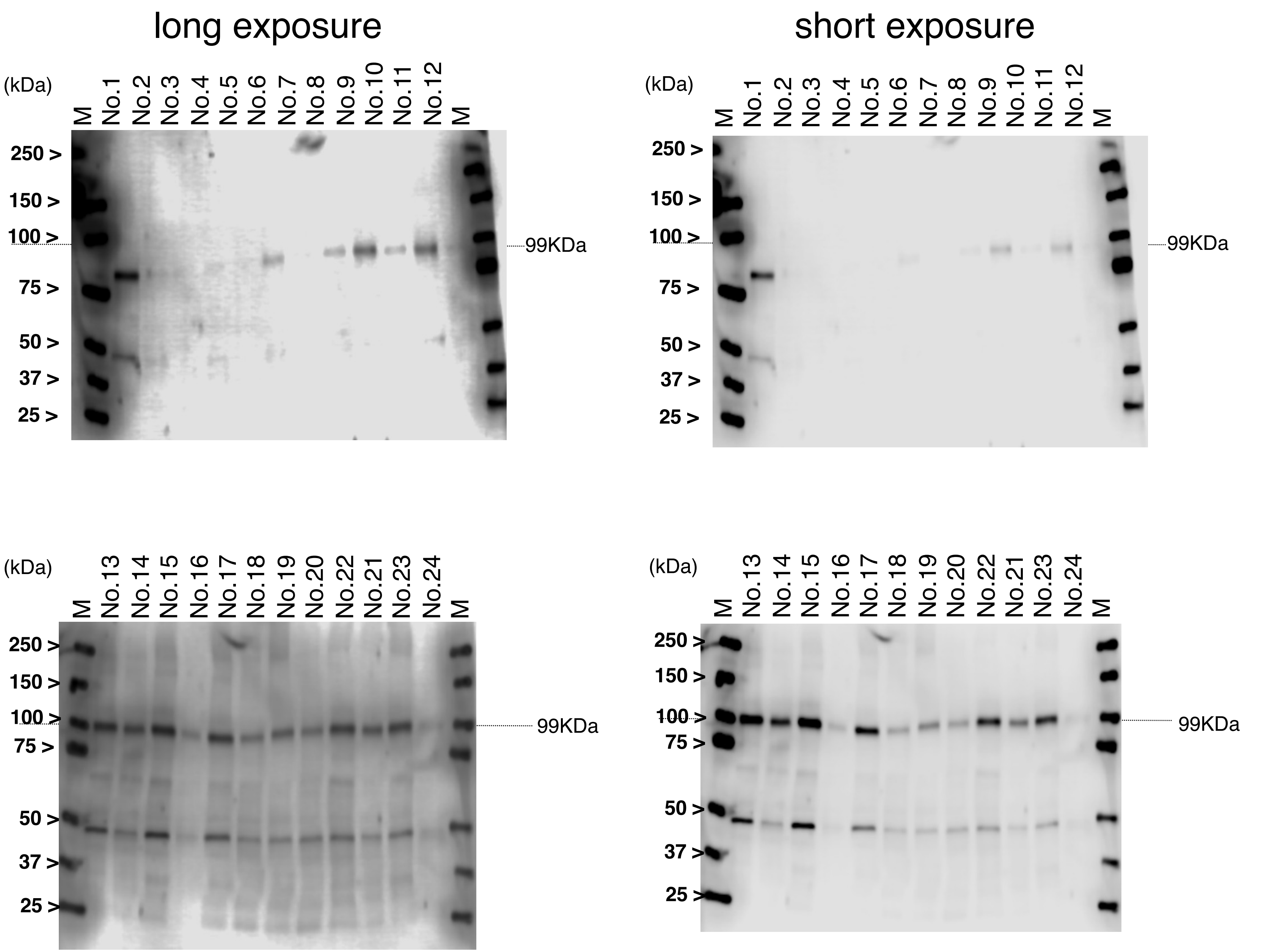

Oota-Ishigaki et al. Source Data

GluA4

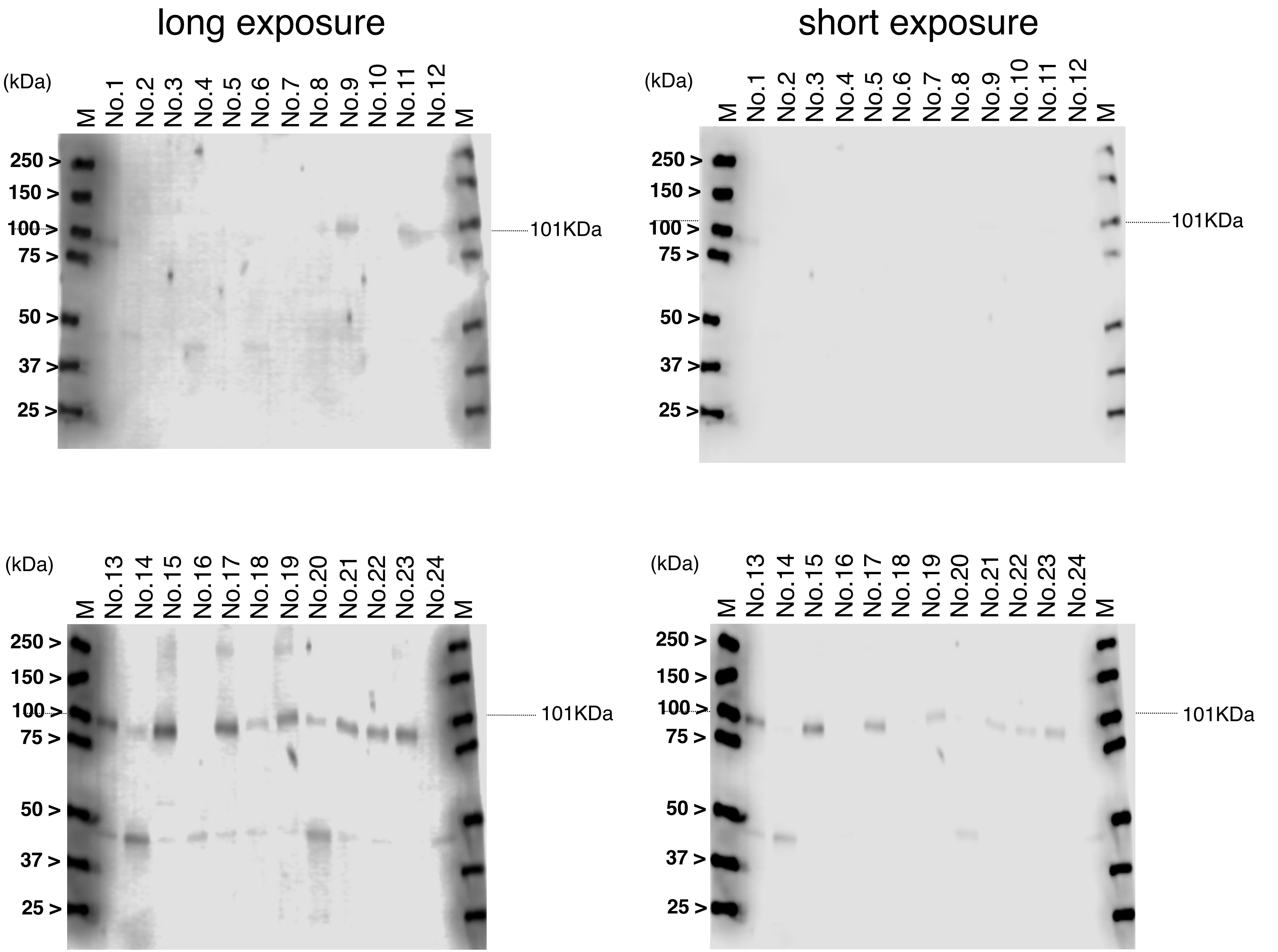

Oota-Ishigaki et al. Source Data

GluA4

(with enhancer)

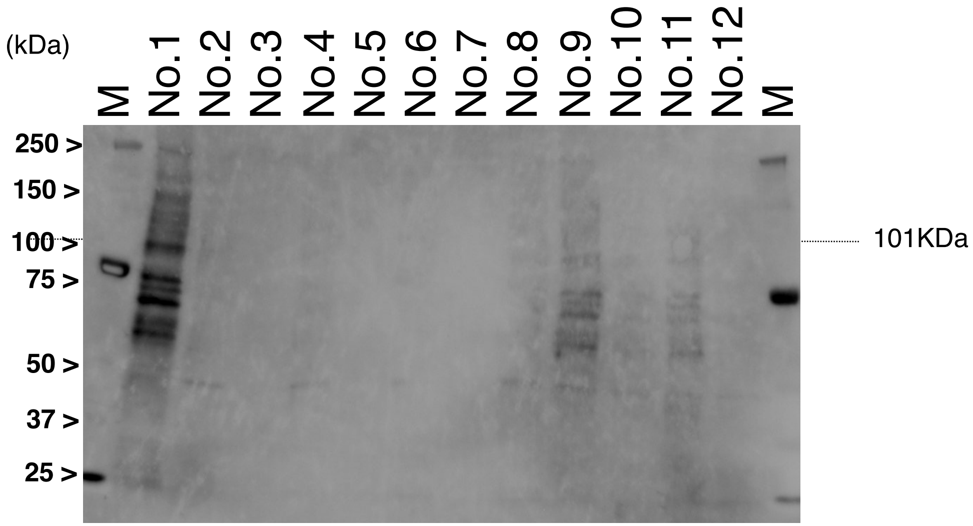

GluN1

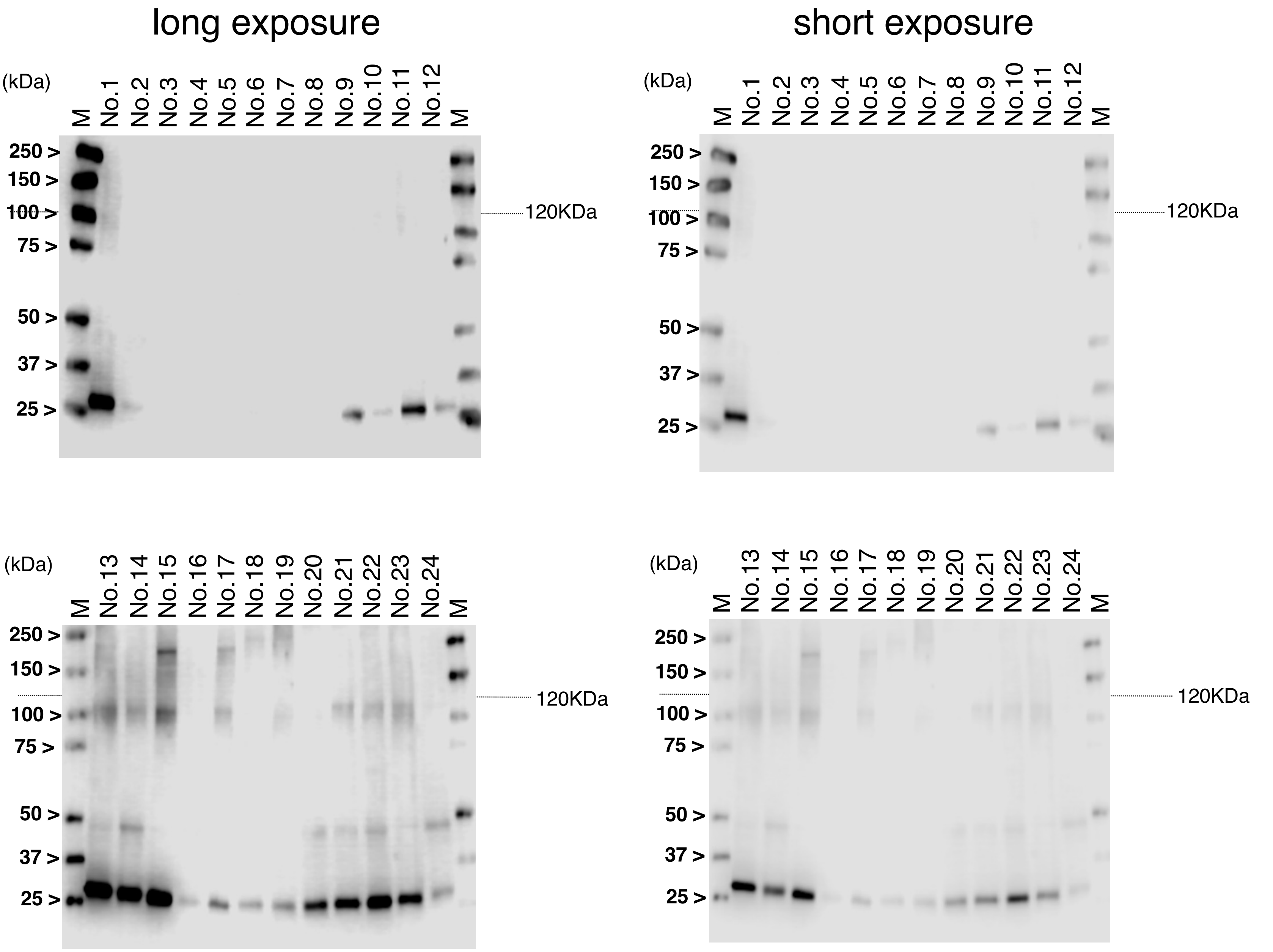

Oota-Ishigaki et al. Source Data

GluN2A

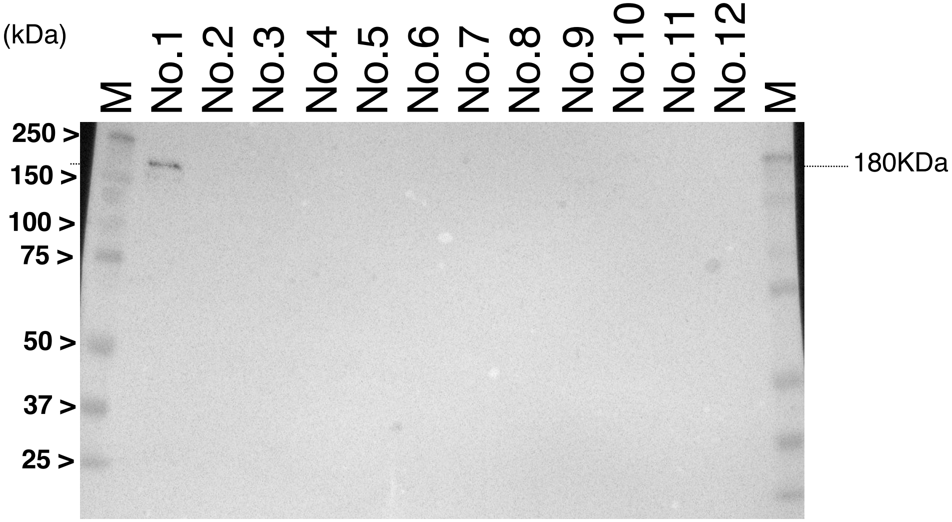

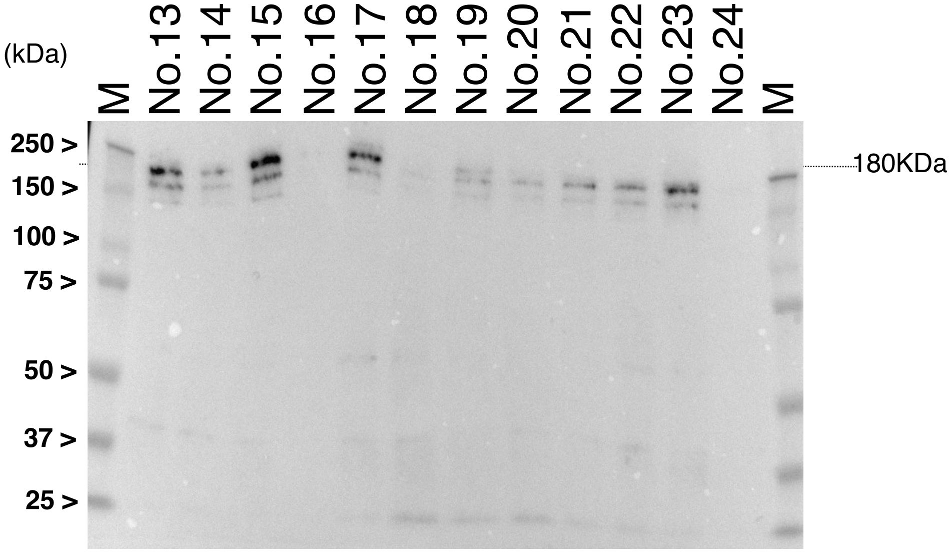

Oota-Ishigaki et al. Source Data

GluN2B

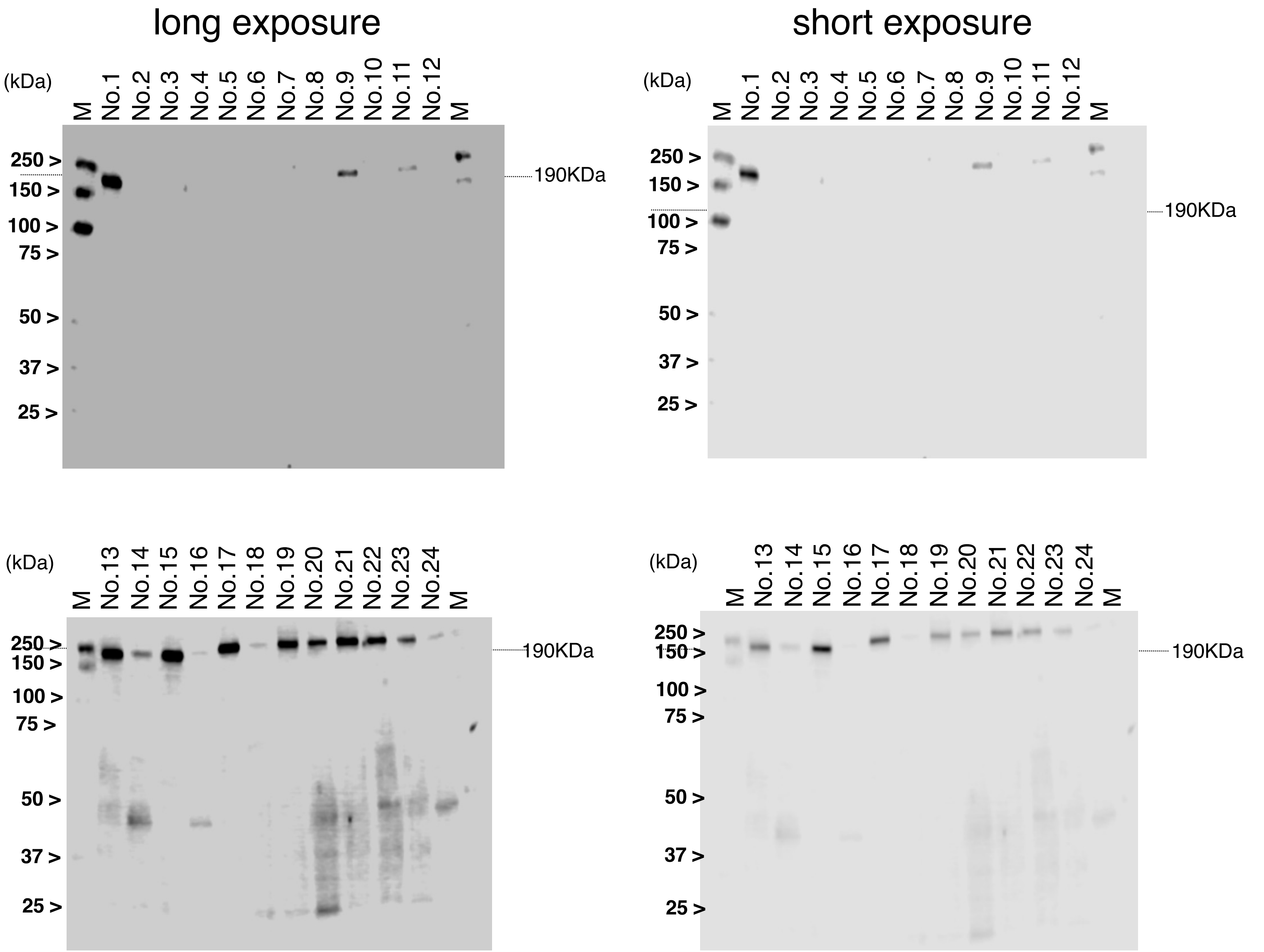

Oota-Ishigaki et al. Source Data

Bassoon

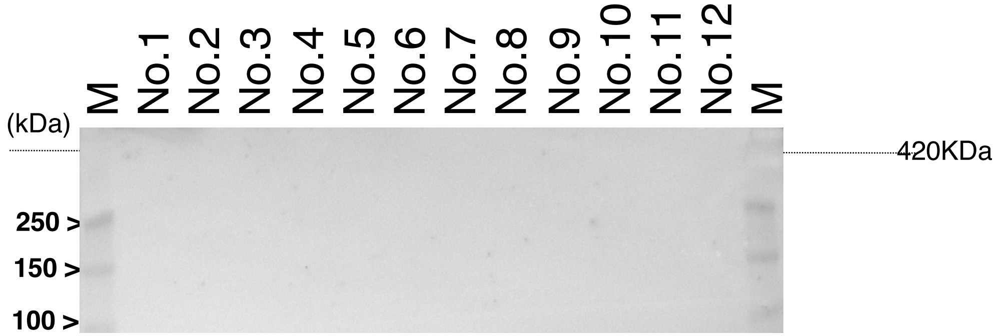

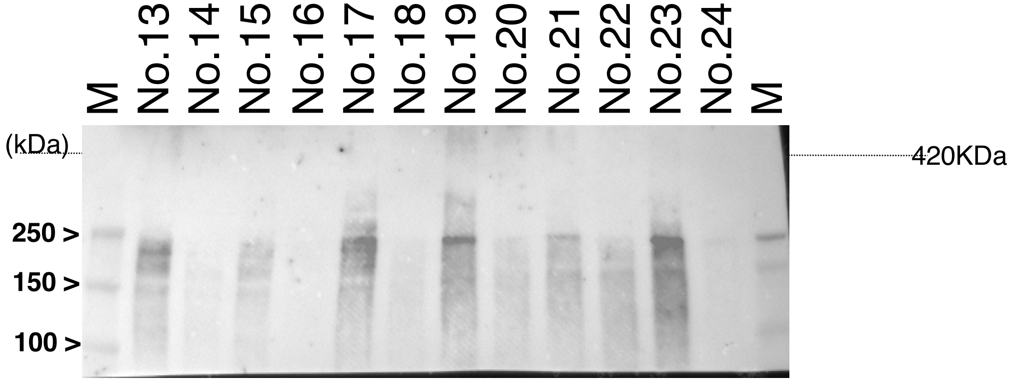

Oota-Ishigaki et al. Source Data

Synaptotagmin-1

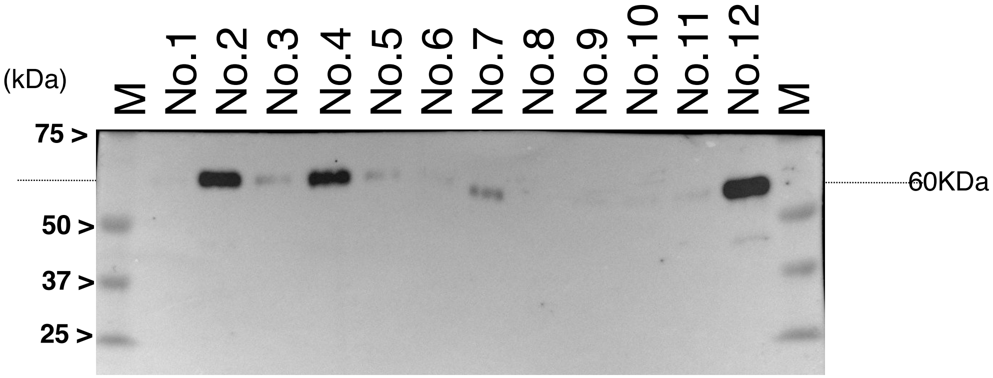

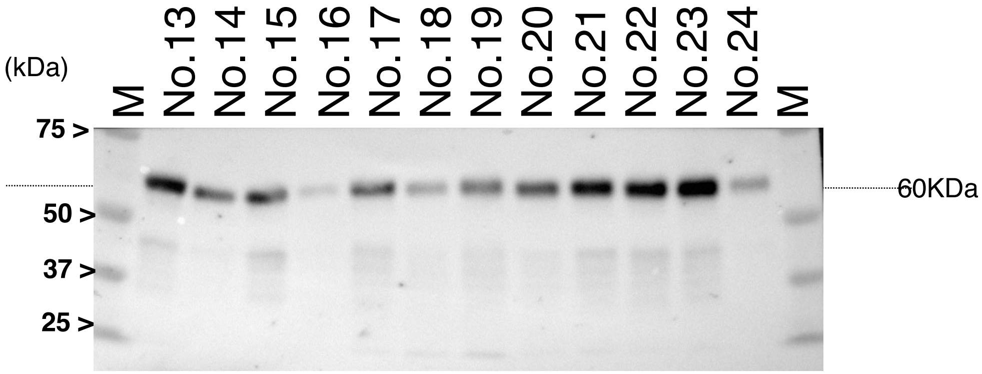

Oota-Ishigaki et al. Source Data

Synaptophysin

(ImmunoStar LD)

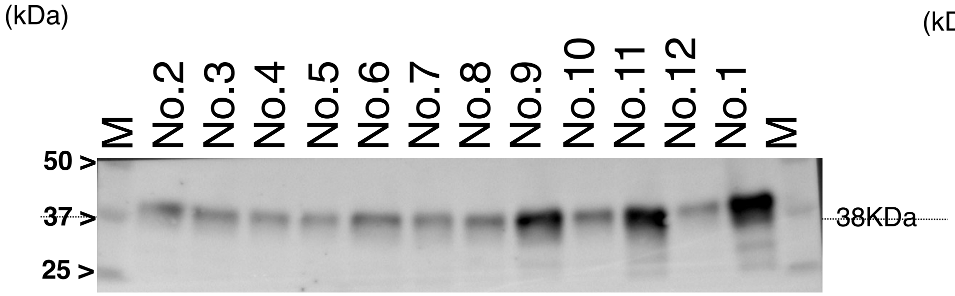

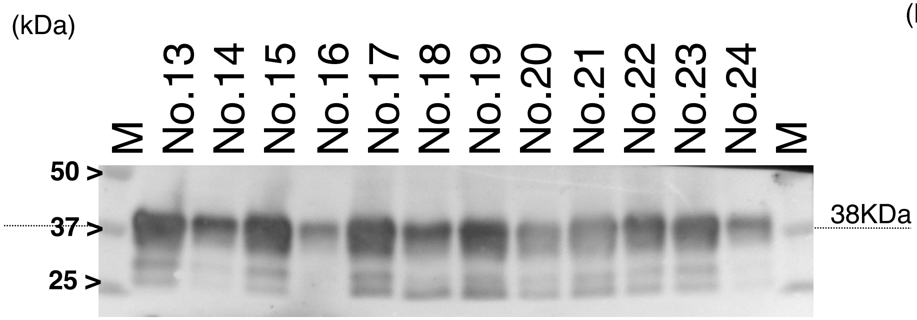

(ECL Prime)

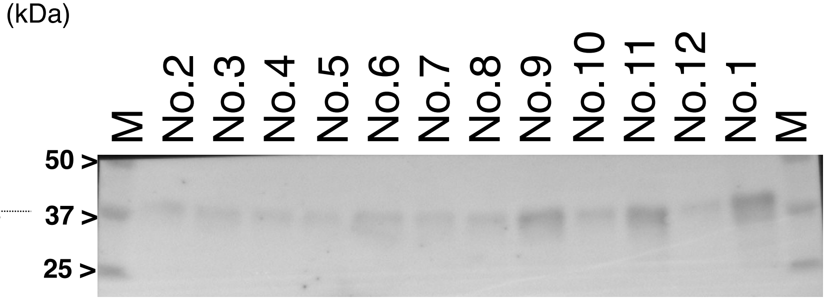

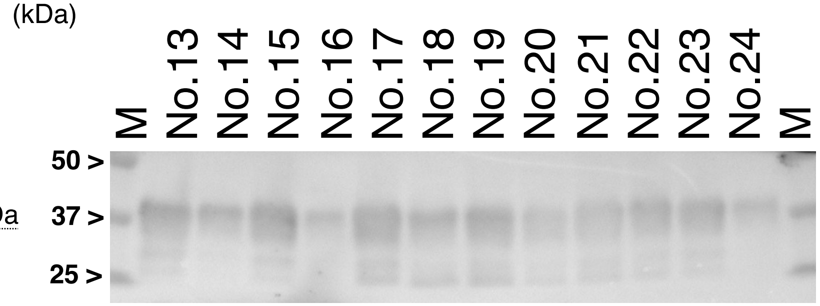

Oota-Ishigaki et al. Source Data

Synapsin-1

(ECL Prime)

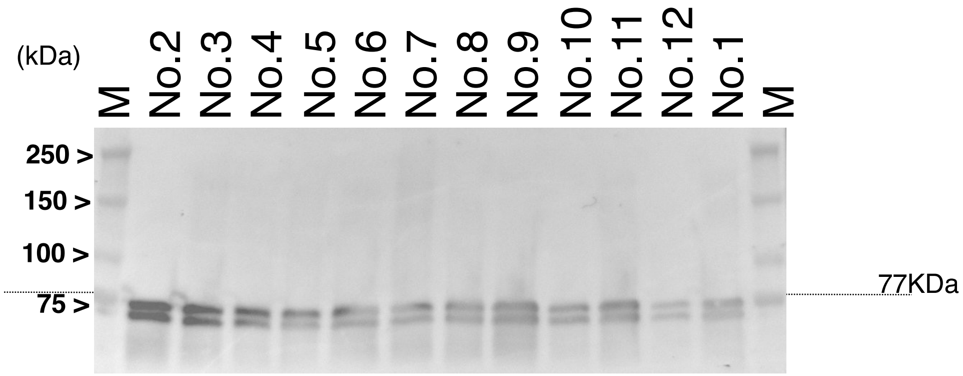

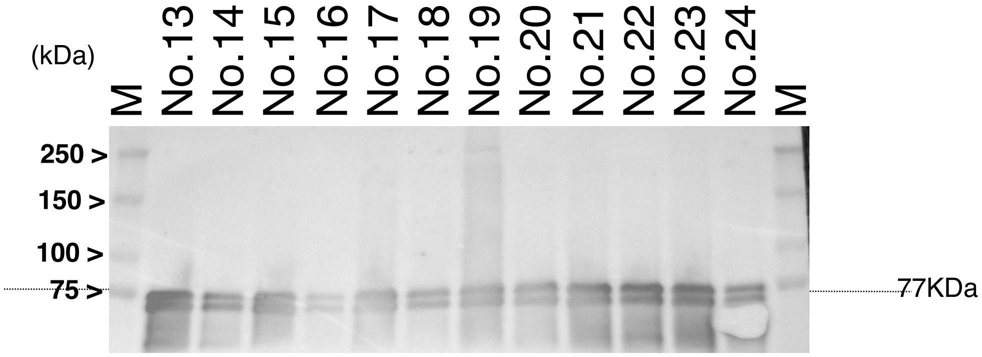

Oota-Ishigaki et al. Source Data

PSD95

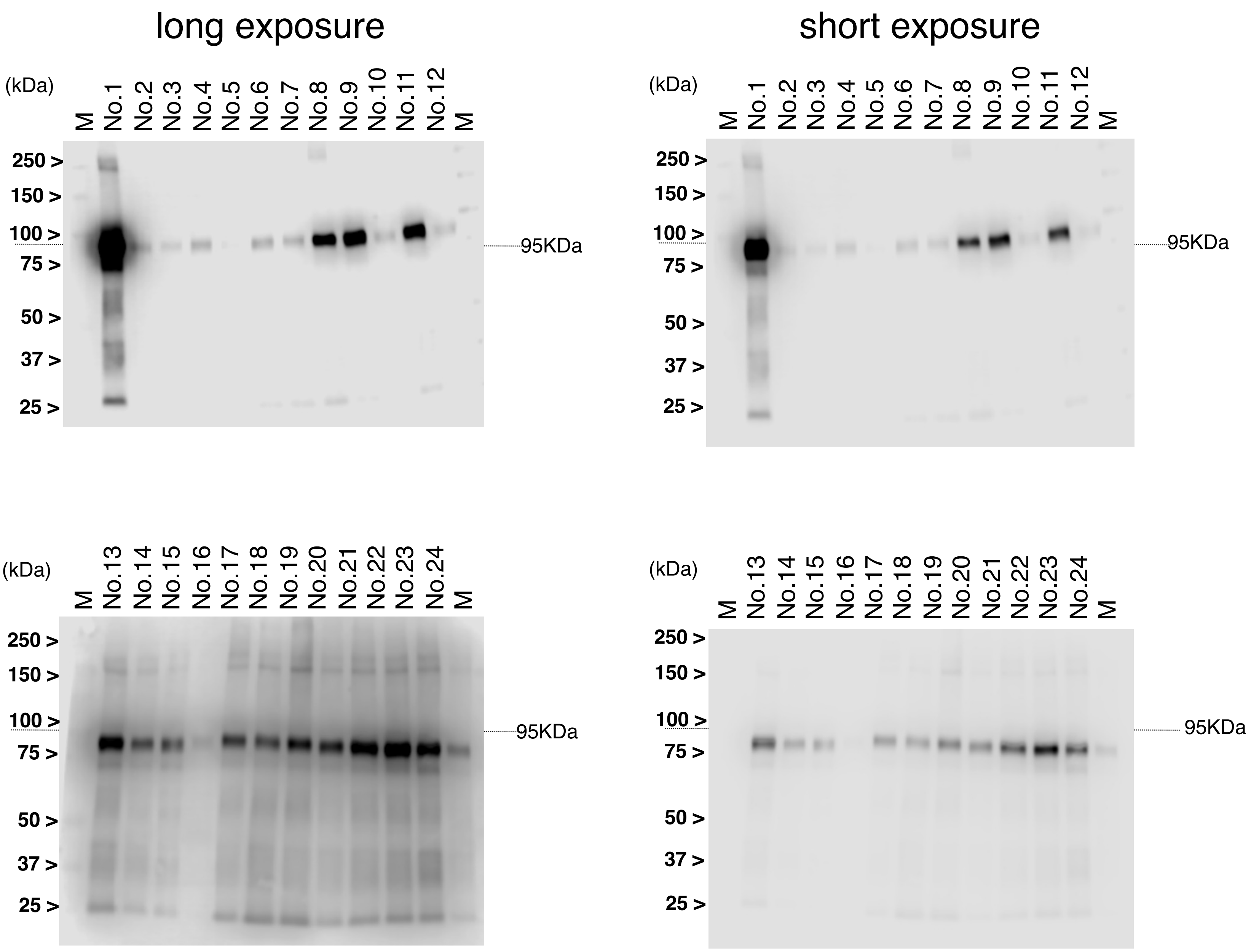

Oota-Ishigaki et al. Source Data

SAP102

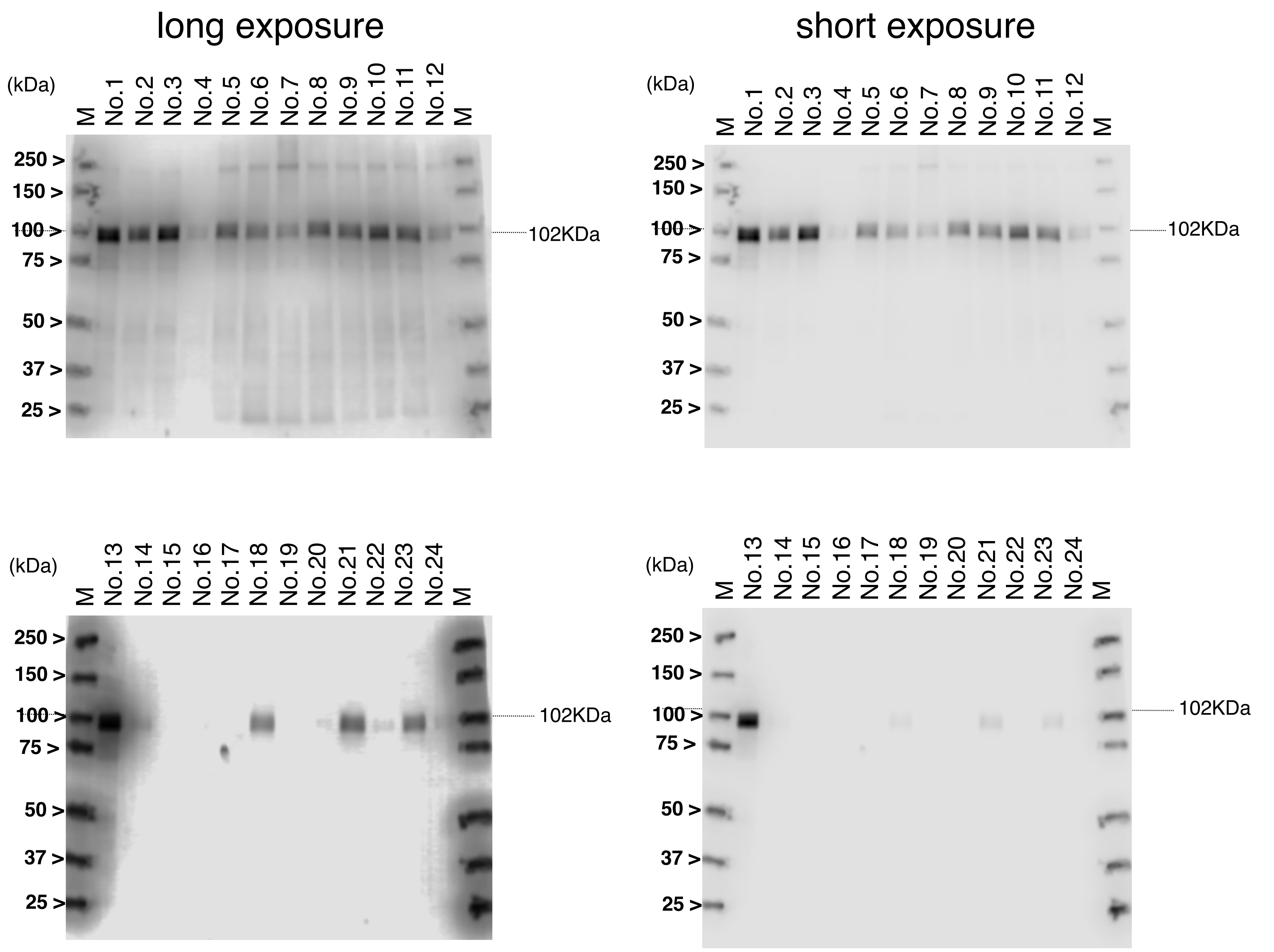

Oota-Ishigaki et al. Source Data

GRIP1

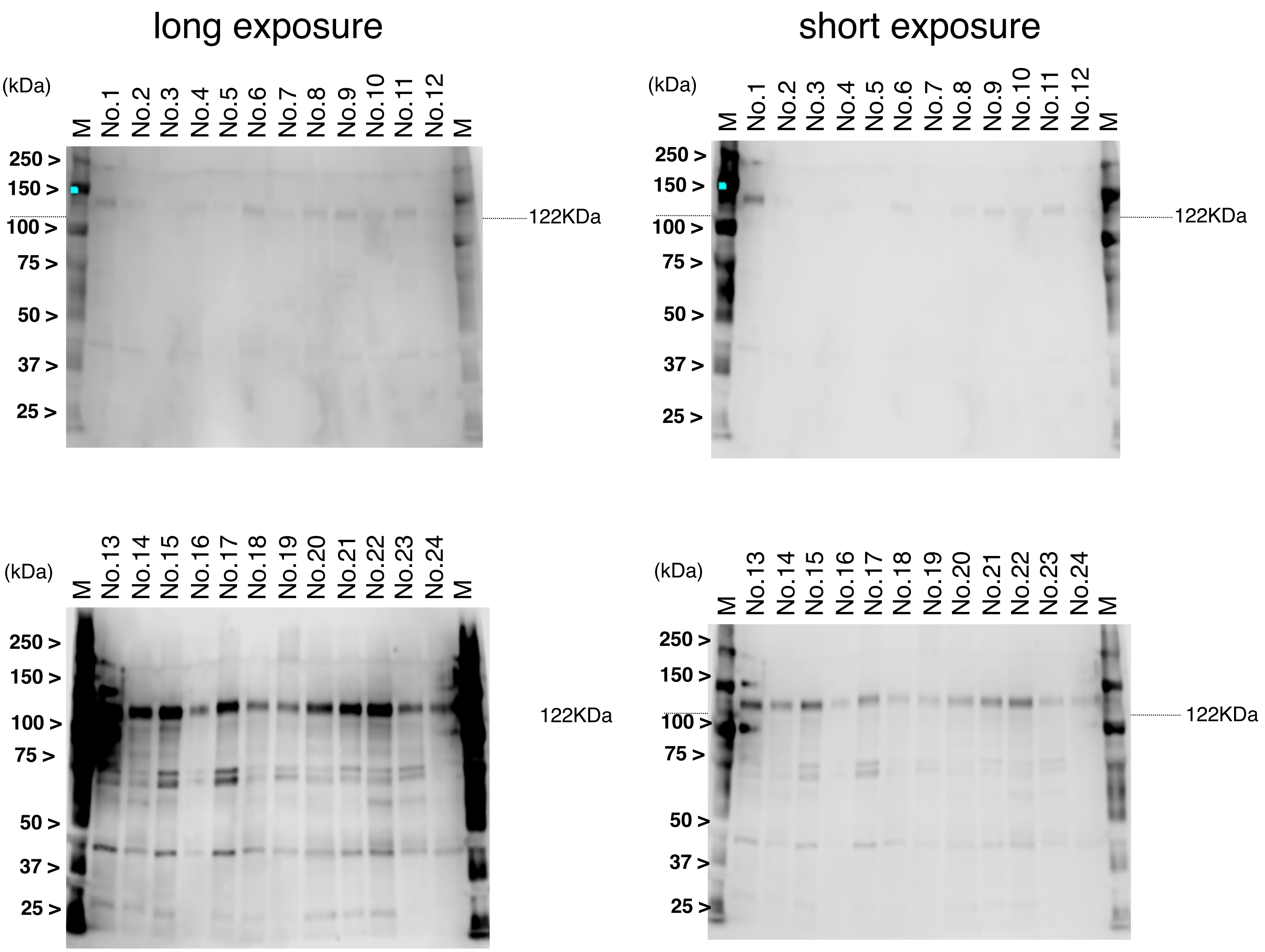

Oota-Ishigaki et al. Source Data

PICK1

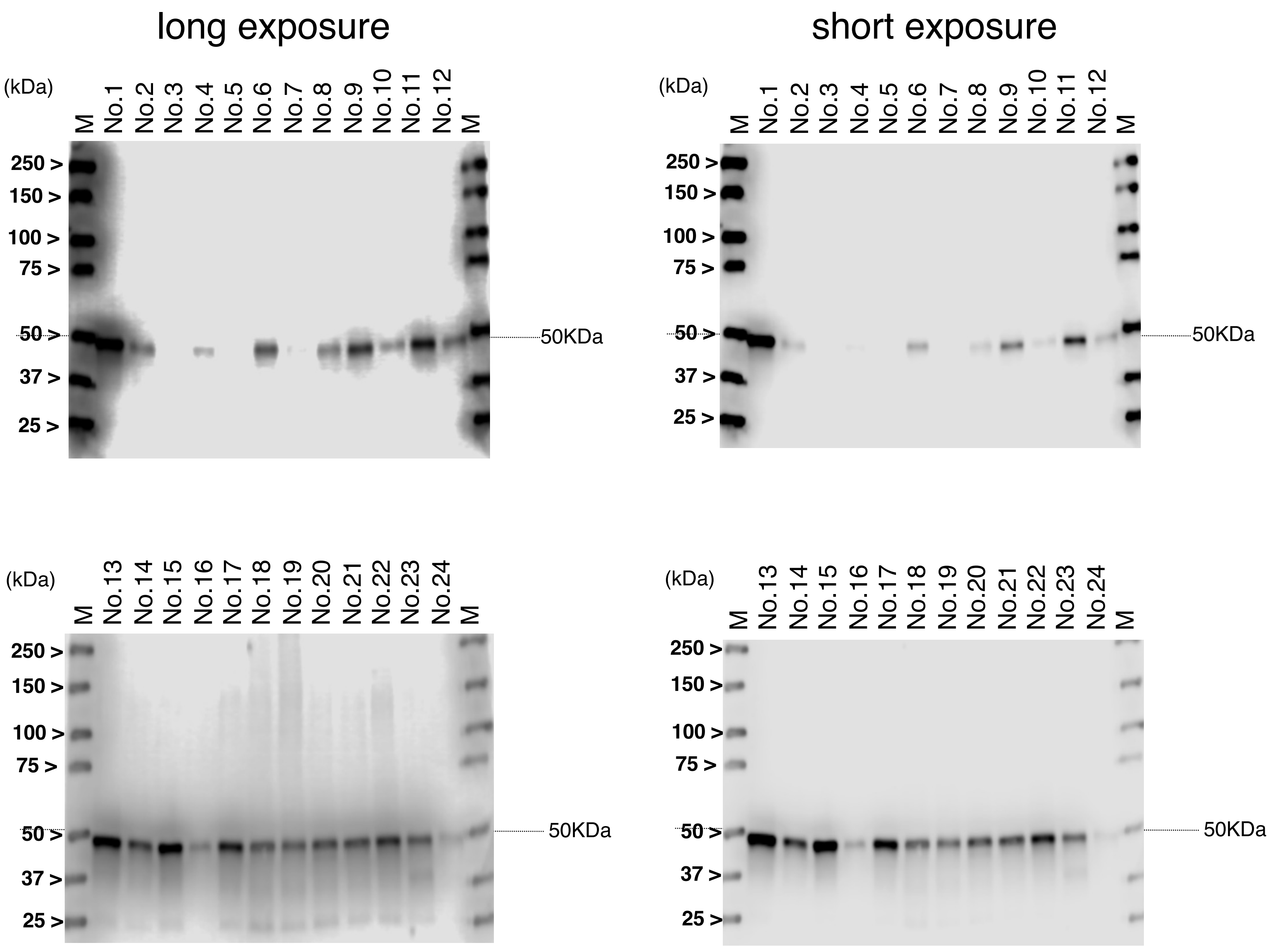

Oota-Ishigaki et al. Source Data

SynGAP

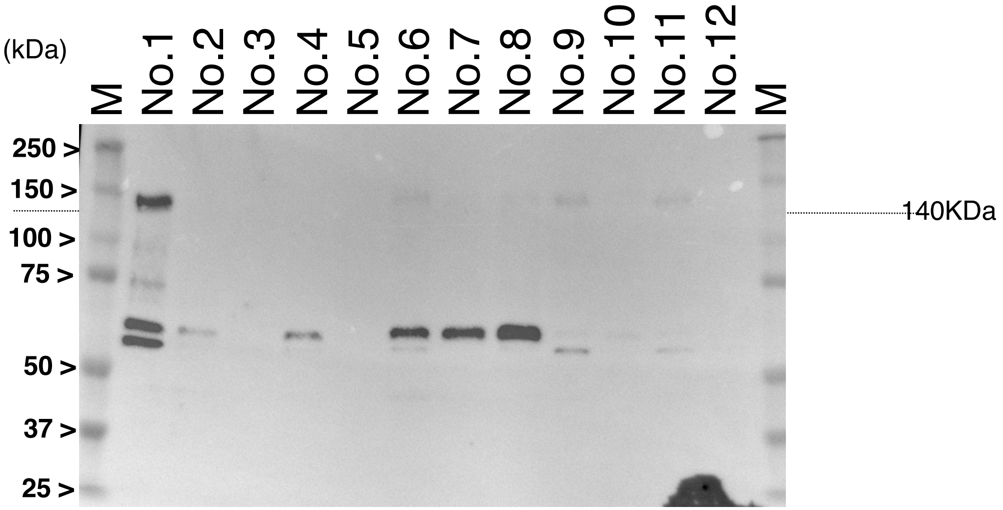
(kDa)
M
No.1
No.2
No.3
No.4
No.5
No.6
No.7
No.8
No.9
No.10
No.11
No.12
M
250 >
150 >
100 >
75 >
50 >
37 >
25 >
140KDa

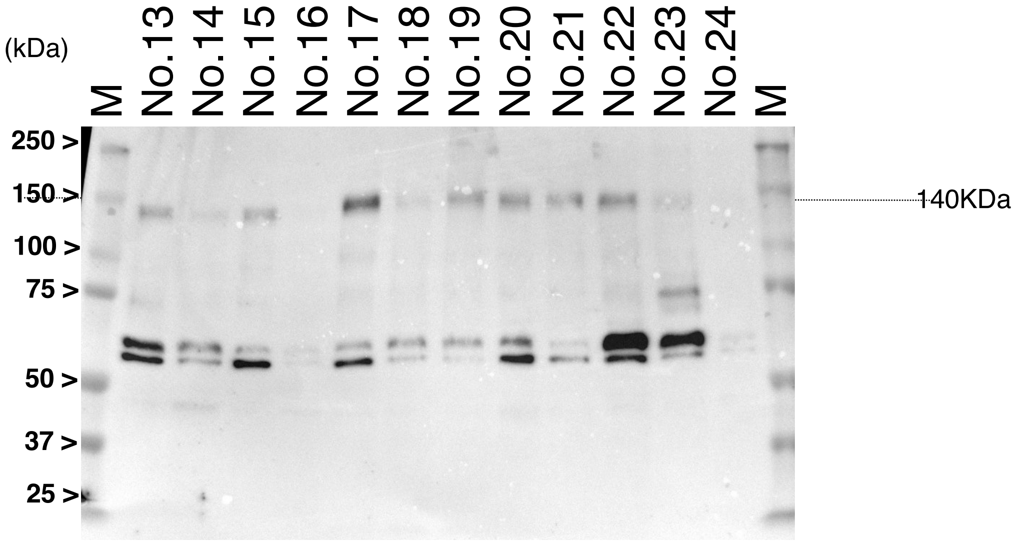
(kDa)
M
No.13
No.14
No.15
No.16
No.17
No.18
No.19
No.20
No.21
No.22
No.23
No.24
M
250 >
150 >
100 >
75 >
50 >
37 >
25 >
140KDa

SHANK3

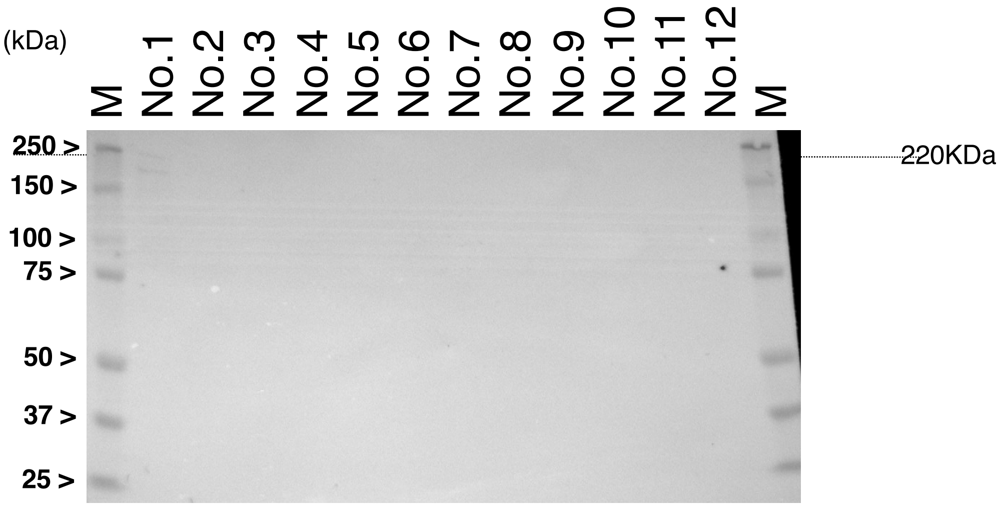

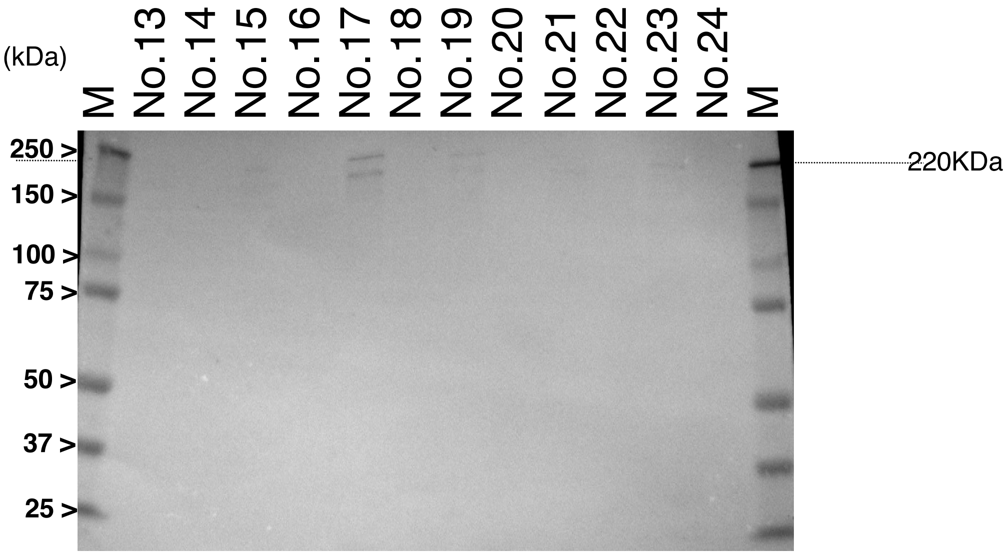

Oota-Ishigaki et al. Source Data

GAPDH

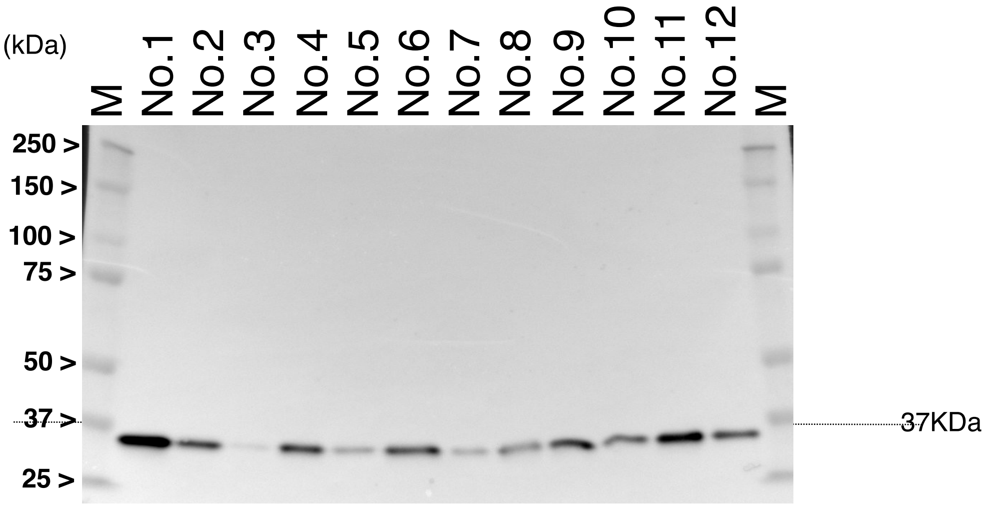

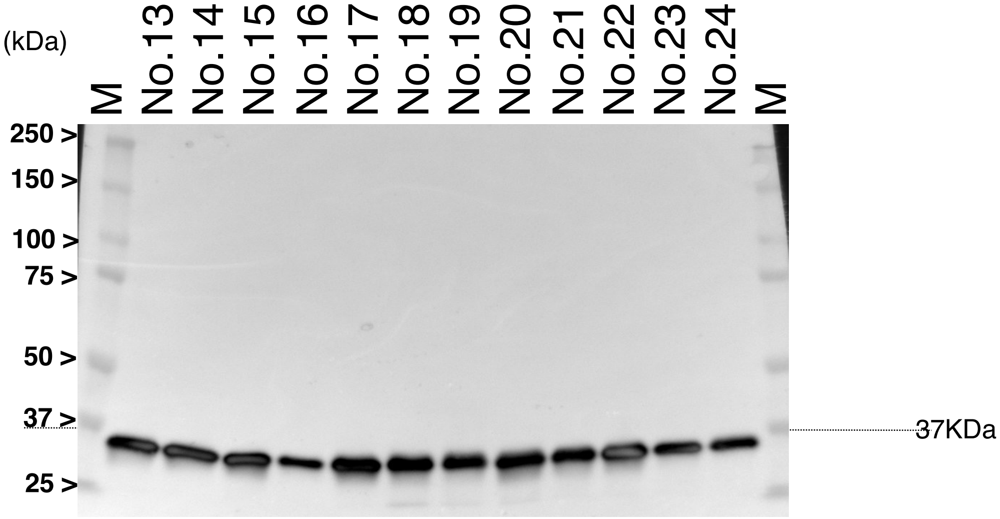

Oota-Ishigaki et al. Source Data

GluA1pS831

(with enhancer)

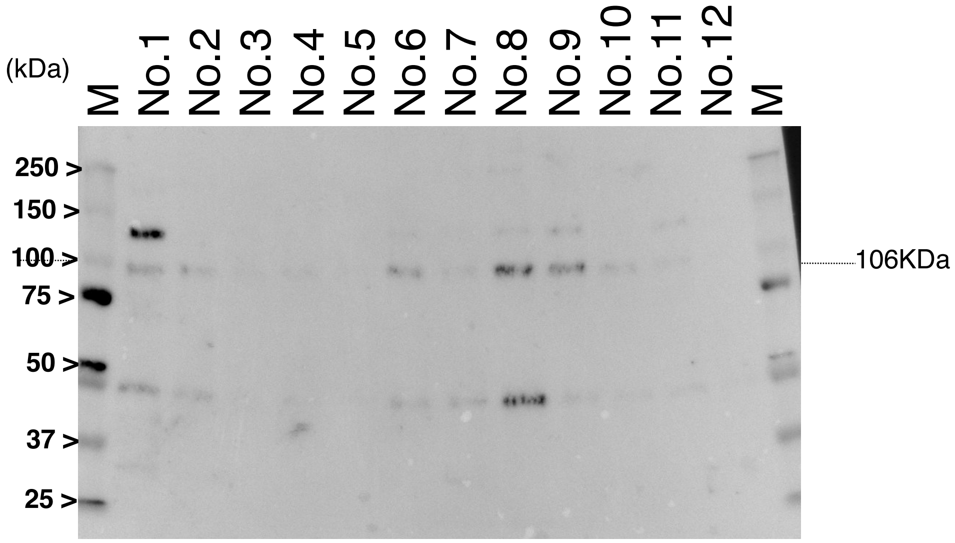

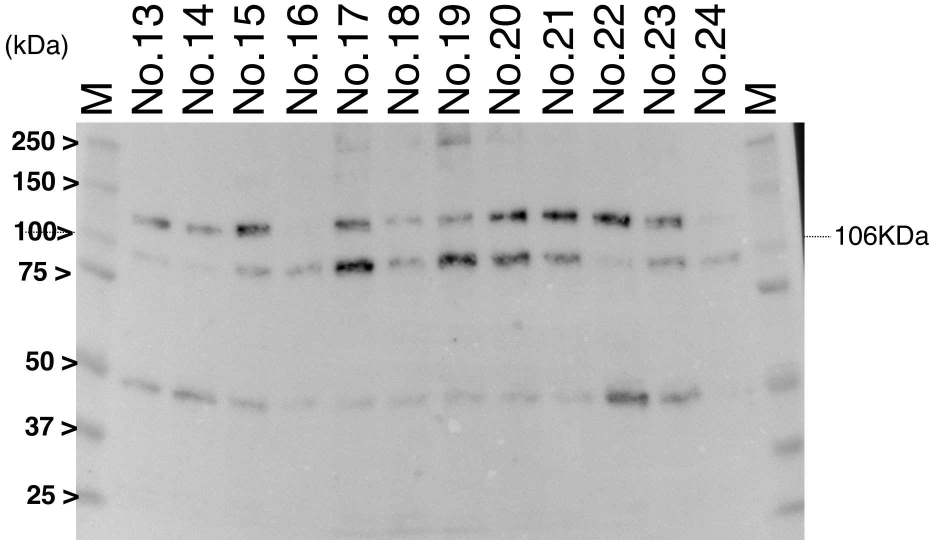

Oota-Ishigaki et al. Source Data

GluA1pS845

(with enhancer)

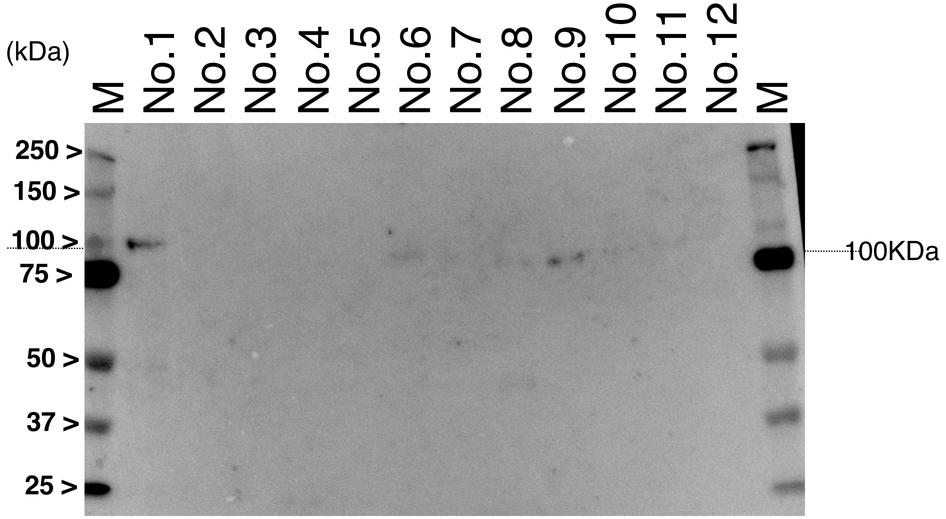

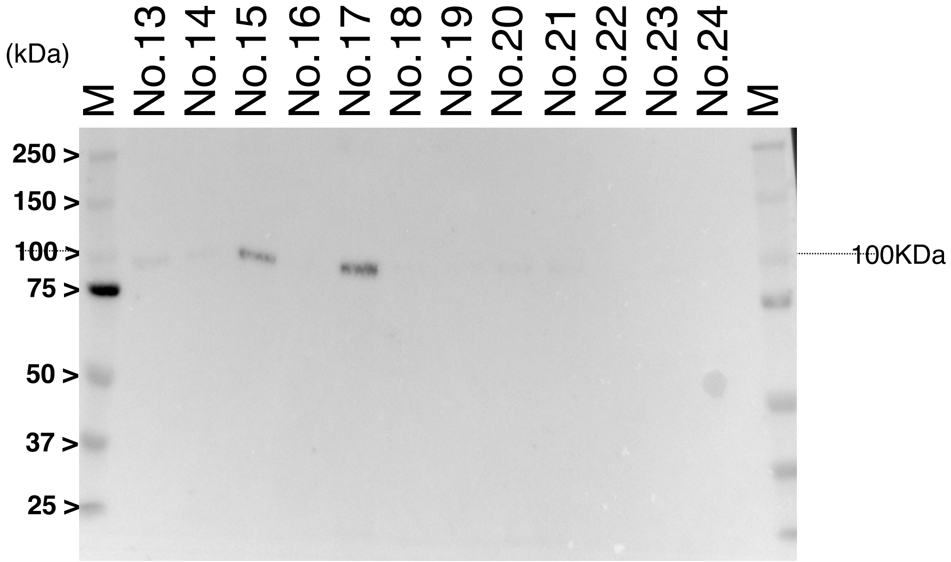

Oota-Ishigaki et al. Source Data

GluA2pS880

(with enhancer)

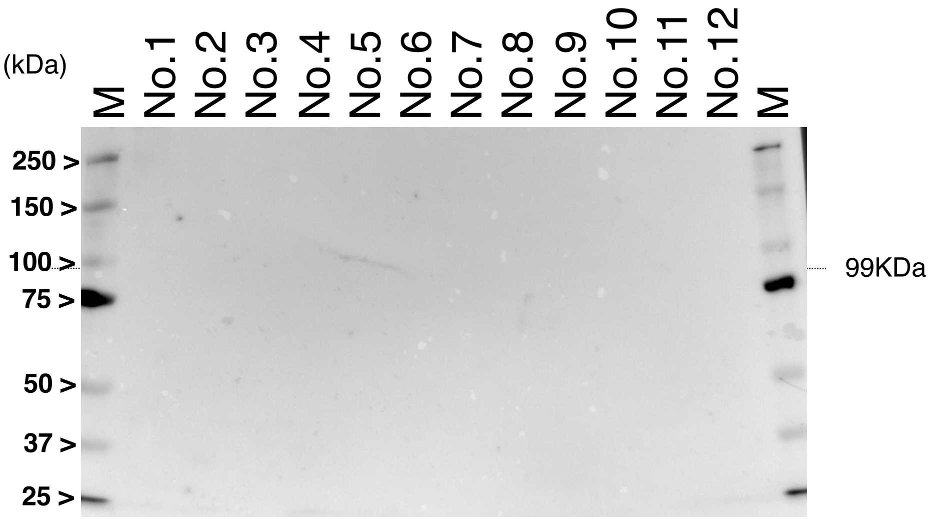

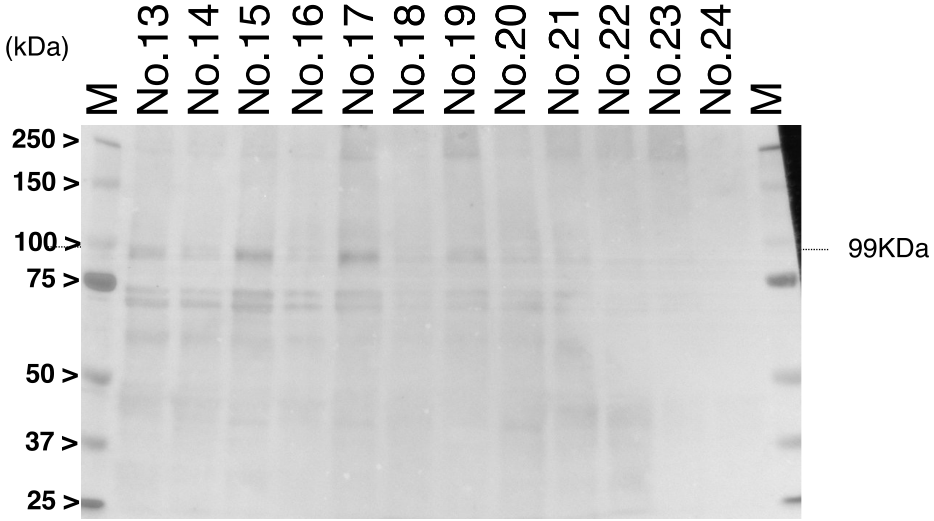

GluA2pY876

(with enhancer)

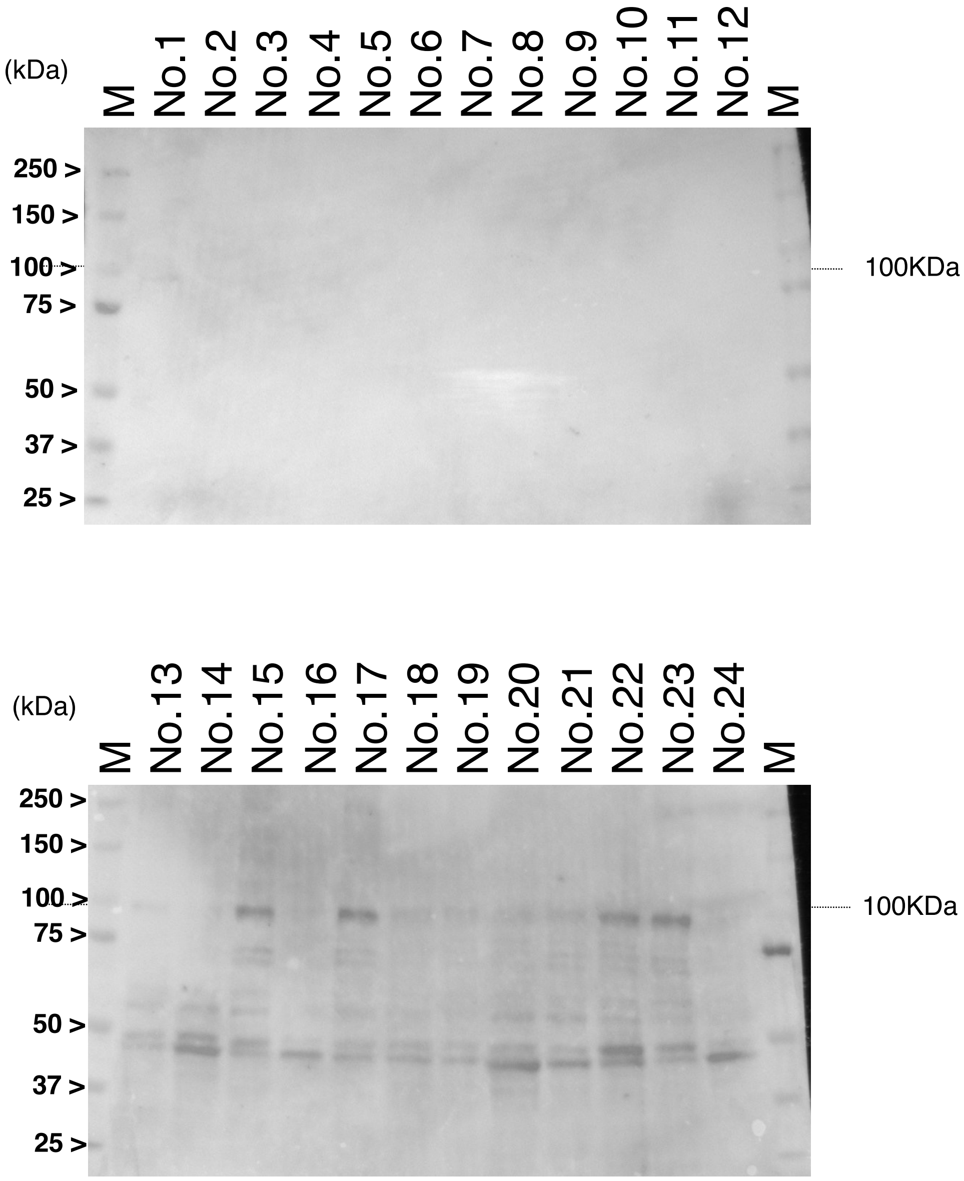

Oota-Ishigaki et al. Source Data

GluN2BpY1472

(with enhancer)

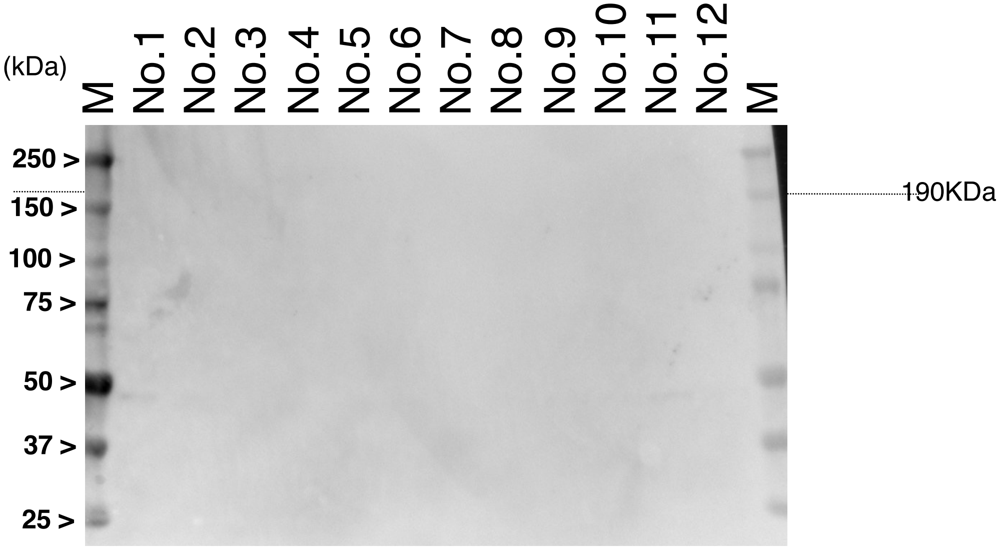

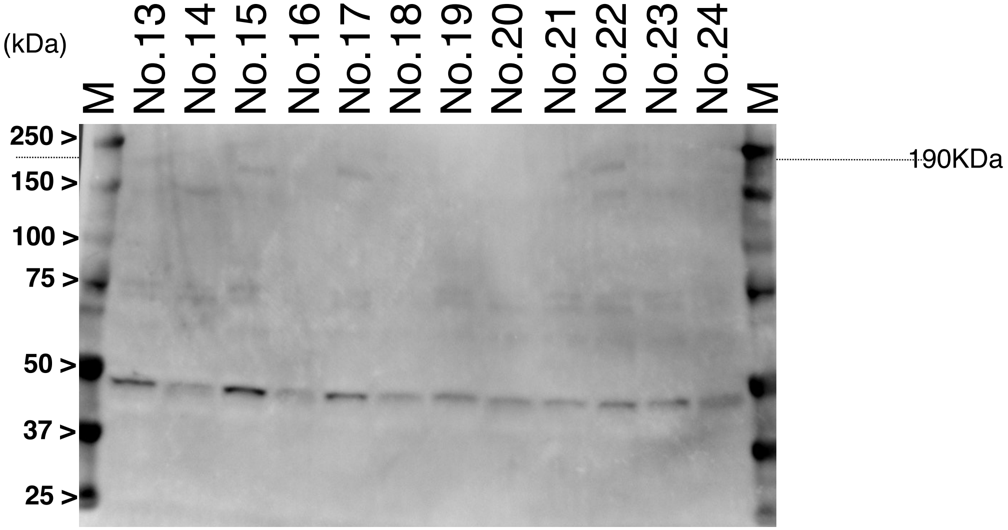

Oota-Ishigaki et al. Source Data
